# Supplementary material for: Barriers and facilitators to implementation of the Ethiopian national cancer control plan strategies: Implications for cervical cancer services in Ethiopia
Source: PLOS Glob Public Health. 2024 Jul 22;4(7):e0003500. doi: 10.1371/journal.pgph.0003500 (PMC11262691; doi:10.1371/journal.pgph.0003500)
Supplement: S1 File — (DOCX) [file pgph.0003500.s001.docx]

**S1 File. Key informants interview guide.**

**Qualitative Research Instruments for Exploring Factors Affecting the National Cancer Control Plan Implementation Framework.**

**Objective 1:** To examine the extent to which the existing institutional strategies for improving quality cervical cancer services support the implementation framework of the national cancer control plan for Ethiopia.

**Guide:** Good morning/afternoon. Interviewer: introduce yourself and ask the key informant to introduce herself/himself. Ensure that the consent form is signed by the participant. Then explain the following questions that will explore the policy implementation challenges to access quality cervical cancer services.

1. **Developing a political commitment to quality cervical cancer prevention and control**

The following questions emphasize identifying the level of political commitment by the national/regional government to improve cervical cancer care.

1. What is your opinion regarding political commitment by the government/national political leadership for cervical cancer care in the country?

2. What do you think about the level of attention given to cervical cancer by activists, cancer societies, citizens, civil society organizations, intellectuals, and patients during the FMoH annual review meeting (ARM) or annual NCDs meeting?

3. What efforts have been made to increase public funding during the health budget allocation process considering the cost-effectiveness of cervical cancer prevention and control services?

1. **Creating transparent and evidence-based priority-setting**

The following questions are intended to identify whether transparent procedures and evidence-based priority-setting approaches exist.

1. What are the measures being taken into account when setting priorities by the government on cervical cancer services? Probe for equity (income, place of residence, and ethnicity).

2. What are the essential cancer medicines, diagnostic tests, or equipment that health facilities would like to have but cannot get funding for?

3. What happens to those who do not receive cervical cancer care for certain conditions because of limitations in public funding?

1. **Strengthening interagency cooperation**

The following questions focus on documenting mechanisms for interagency cooperation and their effectiveness.

1. What are the terms of reference (TOR) of the national cancer committee (NCC)? How effective is the NCC's involvement in goal and target-setting, policy implementation, monitoring, and reporting on cervical CA care in the country? Inquire for the TOR.

2. What specific steps have been taken by the FMoH to mobilize multisectoral assistance of other sectors for the implementation of health in all policies (HiAP) and/or pooled funding?

1. **Integrating evidence into practice**

The following questions explore mechanisms to integrate evidence into medical practice.

1. What is your opinion on the capacity of the unit responsible to develop and review cervical cancer guidelines?
2. What is the process for developing and disseminating cervical cancer clinical guidelines, providers training, and monitoring whether providers adhere to guidelines?
3. How-if at all-are new guidelines incorporated into health professionals’ education and/or continuing education? In practice, who provides the training?
4. **Enhancing population empowerment**

The following questions explore mechanisms and efforts to empower people to be frontline workers for cervical cancer care.

1. What policies, programs, or guidelines are designed to empower communities and service users to take responsibility in identifying their own needs and preferences, and in managing their health with appropriate support from health-service providers, and understanding where cervical cancer services are available?
2. What efforts are being employed to support peer-to-peer/web-based cervical cancer patients including marginalized or vulnerable populations?
3. **Creating the right incentive systems**

The following questions explore current incentive arrangements, how they influence the behavior of the actors in the system, and what impact this may have on the provision or consumption of cervical cancer services.

1. What are the payment rewards to providers for achieving or surpassing the required quality of care? Probe for any performance-based payment (PBP): a fee per beneficiary per month or monthly enhanced cervical cancer care.
2. What are the mechanisms in place to support patients’ adherence to the prescribed medication, peer-to-peer support, transportation cost, or housing to ensure equitable access to facilities?
3. What are the major challenges faced by decision-makers while trying to implement the right incentive systems for providers and/or patients?
