# Supplementary material for: Barriers and facilitators to implementation of the Ethiopian national cancer control plan strategies: Implications for cervical cancer services in Ethiopia
Source: PLOS Glob Public Health. 2024 Jul 22;4(7):e0003500. doi: 10.1371/journal.pgph.0003500 (PMC11262691; doi:10.1371/journal.pgph.0003500)
Supplement: S2 File — (DOCX) [file pgph.0003500.s002.docx]

**S2 File. COREQ checklist**

Consolidated criteria for reporting qualitative studies (COREQ): a 32-item checklist completed for the manuscript on Barriers and facilitators to implementation of the Ethiopian National Cancer Control Plan strategies: Implications for cervical cancer services in Ethiopia.

| **No.** | **Item** | **Guide questions/description** | **Page No.** | **Heading/sub-heading** | **Paragraph No.** |
| --- | --- | --- | --- | --- | --- |
| **Domain 1: Research team and reflexivity** | | | | | |
| Personal Characteristics | | | | | |
| 1. | Interviewer/facilitator | Which author/s conducted the interview or focus group? | 5 | Data collection procedure | 1 |
| 2. | Credentials | What were the researcher's credentials? *E.g. PhD, MD* | 5 | Data collection procedure | 1 |
| 3. | Occupation | What was their occupation at the time of the study? | 5 | Data collection procedure | 1 |
| 4. | Gender | Was the researcher male or female? | 5 | Data collection procedure | 1 |
| 5. | Experience and training | What experience or training did the researcher have? | 5 | Data collection procedure | 1 |
| Relationship with participants | | | | | |
| 6. | Relationship established | Was a relationship established prior to study commencement? | 5 | Data collection procedure | 1 |
| 7. | Participant knowledge of the interviewer | What did the participants know about the researcher? e*.g. personal goals, reasons for doing the research* | 5 | Data collection procedure | 1 |
| 8. | Interviewer characteristics | What characteristics were reported about the interviewer/facilitator? e.g. *Bias, assumptions, reasons and interests in the research topic* | 5-6 | Data collection procedure | 1 |
| **Domain 2: Study design** | | | | | |
| Theoretical framework | | | | | |
| 9. | Methodological orientation and Theory | What methodological orientation was stated to underpin the study? *e.g. grounded theory, discourse analysis, ethnography, phenomenology, content analysis* | 3 | Introduction | 2 |
| Participant selection | | | | | |
| 10. | Sampling | How were participants selected? *e.g. purposive, convenience, consecutive, snowball* | 5 | Sampling | 1 |
| 11. | Method of approach | How were participants approached? e*.g. face-to-face, telephone, mail, email* | 5 | Data collection procedure | 1 |
| 12. | Sample size | How many participants were in the study? | 5 | Data collection procedure | 1 |
| 13. | Non-participation | How many people refused to participate or dropped out? Reasons? | 6 | Data collection procedure | 1 |
| Setting | | | | | |
| 14. | Setting of data collection | Where was the data collected? e*.g. home, clinic, workplace* | 5 | Data collection procedure | 1 |
| 15. | Presence of non-participants | Was anyone else present besides the participants and researchers? | 6 | Data collection procedure | 1 |
| 16. | Description of sample | What are the important characteristics of the sample? *e.g. demographic data, date* | 5-6 | Data collection procedure | 1 & 2 |
| Data collection | | | | | |
| 17. | Interview guide | Were questions, prompts, guides provided by the authors? Was it pilot tested? | 5 | Data collection procedure | 1 |
| 18. | Repeat interviews | Were repeat interviews carried out? If yes, how many? | 6 | Data collection procedure | 1 |
| 19. | Audio/visual recording | Did the research use audio or visual recording to collect the data? | 6 | Data collection procedure | 1 |
| 20. | Field notes | Were field notes made during and/or after the interview or focus group? | 6 | Data collection procedure | 1 |
| 21. | Duration | What was the duration of the interviews or focus group? | 6 | Data collection procedure | 1 |
| 22. | Data saturation | Was data saturation discussed? | 6 | Data collection procedure | 1 |
| 23. | Transcripts returned | Were transcripts returned to participants for comment and/or correction? | 6 | Data processing and analysis | 1 |
| **Domain 3: Analysis and findings** | | | | | |
| Data analysis | | | | | |
| 24. | Number of data coders | How many data coders coded the data? | 6 | Data processing and analysis | 1 |
| 25. | Description of the coding tree | Did authors provide a description of the coding tree? | 6-7 | Data processing and analysis | 1 |
| 26. | Derivation of themes | Were themes identified in advance or derived from the data? | 7 | Data processing and analysis | 1 |
| 27. | Software | What software, if applicable, was used to manage the data? | 7 | Data processing and analysis | 1 |
| 28. | Participant checking | Did participants provide feedback on the findings? | 6 | Data processing and analysis | 1 |
| Reporting | | | | | |
| 29. | Quotations presented | Were participant quotations presented to illustrate the themes/findings? Was each quotation identified? e*.g. participant number* | 7 | Data processing and analysis | 1 |
| 30. | Data and findings consistent | Was there consistency between the data presented and the findings? | 7 | Data processing and analysis | 1 |
| 31. | Clarity of major themes | Were major themes clearly presented in the findings? | 8-12 | Results | 1-14 |
| 32. | Clarity of minor themes | Is there a description of diverse cases or discussion of minor themes? | N/A | N/A | N/A |

N/A: Not applicable

Developed from: Tong A, Sainsbury P, Craig J. Consolidated criteria for reporting qualitative research (COREQ): a 32-item checklist for interviews and focus groups. International Journal for Quality in Health Care. 2007. Volume 19, Number 6: pp. 349 – 357.
