# Supplementary material for: Barriers and facilitators to implementation of the Ethiopian national cancer control plan strategies: Implications for cervical cancer services in Ethiopia
Source: PLOS Glob Public Health. 2024 Jul 22;4(7):e0003500. doi: 10.1371/journal.pgph.0003500 (PMC11262691; doi:10.1371/journal.pgph.0003500)
Supplement: S3 File — (ZIP) [file pgph.0003500.s003.zip › National Cancer Control Plan Data/1. MOH_ transcripts.docx]

**Appendix 3: Qualitative Research Instruments**

- 1. **Exploring Factors Affecting the National Cancer Control Plan Implementation.**

**Objective 1:** To examine the extent to which the Ministry of Health's existing institutional strategies for improving access to and quality of cervical cancer services support the national cancer control plan implementation framework for Ethiopia.

**INTERVIEWER:** Good morning/afternoon. Introduce yourself and ask the key informants to introduce themselves. Ensure that the consent form is signed by individual participants. Then explain the following questions that will explore the national cancer control plan implementation challenges to access quality cervical cancer services.

1. **Developing a political commitment to quality cervical cancer prevention and control**

The following questions aim to understand the level of political commitment by the national/regional government to improve cervical cancer care.

1. What is your opinion regarding political commitment by the government/national political leadership for cervical cancer care in the country?

- There was a high government commitment to the leadership and guidance of the First Lady. The MOH established cervical cancer prevention and control program with the allocation of adequate personnel to run the program. An adequate budget was allocated for cervical cancer screening and treatment services. The government allocated an ETB400 million budget for cervical cancer screening and treatment services in the country. This was done in coordination with partners such as the Centers for Disease Control and Prevention (CDC) support for HIV-positive women. With the strong commitment of the government, 1218 health facilities in 800 woredas (districts) of the country started to provide cervical cancer screening and treatment services. at least one health facility per district provides cervical cancer (Cx Ca) services.

2. What do you think about the level of attention given to cervical cancer by activists, cancer societies, citizens, civil society organizations, intellectuals, and patients during the FMOH annual review meeting (ARM) or annual NCDs meeting?

- The cervical cancer (Cx Ca) annual review meeting was done with the involvement of regional and zonal focal persons for the evaluation of cervical cancer services. The MOH also conducted cervical cancer Zoom meetings with zonal offices every month for evaluation of combat cervical cancer (3Cs) services. The 3Cs' performance was evaluated in the presence of regional focal persons. The MOH implemented 3Cs service at cervical cancer screening and treatment facilities that aimed to screen 5 women in a day at a given health facility. Accordingly, a total of 367, 000 women were screened from July 2021 to June 2022.

3. What efforts have been made to increase public funding during the health budget allocation process considering the cost-effectiveness of cervical cancer prevention and control services?

- The government allotted millions of Ethiopian Birr and secured partners' support for some of the cervical cancer prevention and control services. This multiple stakeholders' involvement to combat cervical cancer in the country resulted in the cost-effectiveness of cervical cancer prevention and control services. Women aged 30-49 were obtaining 100% free screening and treatment services at public health facilities.

1. **Creating transparent and evidence-based priority-setting**

The following questions are intended to identify whether transparent procedures and evidence-based priority-setting approaches exist.

1. What are the measures being taken into account when setting priorities by the government on cervical cancer services? Probe for equity (income, place of residence, and ethnicity).

- The government put a huge investment in the prevention and control of cervical cancer at primary, secondary, and tertiary levels. Income, place of residence, and ethnicity were taken into consideration when setting priorities by the government on cervical cancer services in the country.
- **For primary prevention**, the MCH department/MOH leads an HPV vaccine immunization program where two doses were given to school girls by the age of 15. It was required to be given as one of the routine immunization programs.
- The NCDs including cervical cancer public awareness in the community were found to be inadequate as health extension workers (HEWs) were required to receive integrated refresher training. The training to HEWs was not given in full as it was projected to cost over ETB400 million. Therefore, the training was focused on urban settings but not on rural ones. However, this was planned to be done by the MOH.
- **The secondary prevention** was focused on the see-and-treat approach (STA) with visual inspection using acetic acid (VIA) and treatment with cryotherapy and thermal ablation mainly for women aged 30-49 in 1218 health facilities, at least one per woreda nationwide. The focus was on this age group as it could result in significant cervical cancer screening and treatment outcomes. In Ethiopia, 10.7 million women (aged 30-49) were eligible for cervical precancer screening. As of August 2022, 900, 000 women were screened which was 8.4% of the 10.7 million targets.
- **Tertiary prevention** was aiming to increase the number of tertiary-level hospitals providing chemotherapy, radiotherapy, and radical surgery services. The MOH established cervical cancer screening and treatment centers of excellence in the regions including Tikur Anbessa, Saint Paul, Jimma, Adama, Bahir Dar, Gondar, Dessie, Hawassa, and Arba Minch Specialized Hospitals.
- **Cancer registry-** Up to 5 comprehensive care oncology centers were expected to start paper-based cancer registry services such as Jimma and Haramaya specialized university hospitals. The Addis Ababa City Cancer Registry (AACCR) was the first population-based cancer registry in Ethiopia. It was collecting cancer diagnostic and/or treatment services from 17 selected health facilities in Addis Ababa. The cancer registries would serve as a source of data for the forecasting and quantification of cancer medicine need.
- **LEEP** was done at 50 hospitals in the country. In Addis Ababa, seven hospitals were providing LEEP service including Tikur Anbessa, St. Peter, Gandhi, Zewditu, Tirunesh Beijing, Yekatit/Abebech Gobena, and St. Paul hospitals.
- **Pap smear** was not available in many health facilities. So, they had to refer clients to the Family Guidance Association (FGA) of Ethiopia and/or private diagnostic facilities. The Pap smear test was required to be done mainly for women aged >50 by pathologists.
- **Radiotherapy-** During this study radiotherapy service was given at Tikur Anbessa, Jimma, and Haramaya specialized hospitals. This service was expected to expand to 6 regional hospitals including Hawassa, Mekele, Gondar, and St. Paul Hospitals.
- **HPV DNA test-** Partners such as CDC allocated a budget to support HPV DNA tests for HIV+ women at 70 health facilities in 5 regions. Expansion of the test is expensive so the focus was on screening with VIA.
- **Cancer medicines-** The MOH allocated ETB156 million per year for the procurement of cancer medicines. The cancer treatment hospitals provided services by oncologists and were required to forecast and quantify their cancer medicines. The Ethiopian Pharmaceuticals Supply Service (EPSS) procures and distributes them based on their requisitions. The unavailability of cancer medicines could be an issue of poor forecasting and quantification by the chemotherapy centers in hospitals and/or long procurement lead times encountered by EPSS. Moreover, chemotherapy for breast cancer was provided in 16 hospitals working with clinical oncologists.
- **Center of excellence:**  The MOH established 8 centers of excellence in the country including Tikur Anbessa, Saint Paul, Jimma, Adama, Hawassa, Arba Minch, Bahir Dar…
  **The WHO 90-70-90 targets:** To achieve this target, the MOH implemented the Cx Ca 3Cs initiative in 1218 public health facilities in the country. Due to this initiative, the ministry was able to screen 367, 000 women in 2021/22, double the screening done in 2020/21. So far 900, 000 women were screened out of the eligible 10.7 million equals 8.4%, which is way below the WHO’s 70% target for screening.

2. What are the essential cancer medicines, diagnostic tests, or equipment that health facilities would like to have but cannot get funding for?

- There was inadequate availability of generic cancer medicines and one radiotherapy center in the city, Tikur Anbassa Hospital. This was influenced by resources constraint.

3. What happens to those who do not receive cervical cancer care for certain conditions because of limitations in public funding?

- When cancer medicines were not available at public facilities clients were forced to procure them from private pharmacy outlets. While for radiotherapy, clients had to travel overseas or wait for months to get the service. During this study, additional radiotherapy centers were established at Jimma University (southwestern Ethiopia) and Haramaya University (eastern Ethiopia) Specialized Hospitals in the country.

1. **Strengthening interagency cooperation**

The following questions focus on documenting mechanisms for interagency cooperation and their effectiveness.

1. What are the terms of reference (TOR) of the national cancer committee (NCC)? How effective is the NCC's involvement in goal and target-setting, policy implementation, monitoring, and reporting on cervical CA care in the country? Inquire for the TOR.

- The TWG was established for cervical cancer prevention and control including MOH, hospitals, universities, partners, and others. The TWG was multisectoral and meets every 3 months a year. The TWG i) develops and revises guidelines and strategies including cervical cancer prevention and control guidelines, and mentorship guidelines; ii) adopts guidelines; iii) program implementation and monitoring; iv) works on awareness of women in different sectors. The DHIS2 M and E indicators include screening rate, positivity rate, and treatment rate. The quality of the Cx Ca services was monitored by the MOH, regional, and zonal health offices at 200 health facilities in the country using the mentorship guidelines. Partners support these activities.

1. What specific steps have been taken by the FMOH to mobilize multisectoral assistance of other sectors for the implementation of health in all policies (HiAP) and/or pooled funding?

- The development partners work with the MOH. Allocate funds to support various areas including HPV DNA tests, Pap smear tests… For improving the uptake of the HPV vaccine uptake, the MOH works with the Ministry of Education, the Ministry of Youth and Women Affairs, women's forums… The MOH strategy encourages public-private partnership (PPP) especially in the provision of cancer services though it was required to be strengthened in various Cx Ca service provision areas.

1. **Integrating evidence into practice**

The following questions explore mechanisms to integrate evidence into medical practice.

1. What is your opinion on the capacity of the unit responsible to develop and review cervical cancer guidelines?

- There was an adequate national capacity to develop and review cervical cancer guidelines. The TWG members were responsible to develop and review cervical cancer guidelines. The members were from MOH, CDC, ICAP, WHO, Pathfinder International, St. Paul Millennium Medical College, Addis Ababa University/College of Health Sciences, and other hospitals. The members develop training materials, Cx Ca prevention and control guidelines, Cx Ca mentorship guidelines, M & E tools, and IEC and SBCC materials.

1. What is the process for developing and disseminating cervical cancer clinical guidelines, providers training, and monitoring whether providers adhere to guidelines?

- The TWG brainstorms, refer to global guidelines, prepares zero drafts, reviews the drafts, and submits the draft to the minister of health. The minister provides feedback and incorporates the inputs, and launches and adopts the guidelines. The training of trainers (TOT) was provided to regions and the regions cascade the basic training. The MOH, regions, and zonal offices monitor the implementation of guidelines in monthly, quarterly, and annual meetings. Additionally, there were mentorship and supportive supervision work.

1. How-if at all-are new guidelines incorporated into health professionals’ education and/or continuing education? In practice, who provides the training?

- MOH provided TOT training for regional oncologists. The regions cascade basic training on identified gaps. There were 2-3 basic training hubs staffed with at least two gynecologists and 2 middle-level BSc nurses. The hubs were in well-established hospitals and focused on improving skills and refresher training. The training materials were prepared from the cervical cancer prevention and control guidelines and provided by university hospitals.

1. **Enhancing population empowerment**

The following questions explore mechanisms and efforts to empower people to be frontline workers for cervical cancer care.

1. What policies, programs, or guidelines are designed to empower communities and service users to take responsibility for identifying their own needs and preferences, managing their health with appropriate support from health-service providers, and understanding where cervical cancer services are available?

- With support from the MOH and its partners, the national audio/radio and video/television messages on cervical cancer prevention and control services were transmitted for 3 months, 4 times per week. There were inadequate radio and television messages transmitted at Addis Ababa. The city was expected to reach more population through available FM radio channels and Addis Media Network Television. This requires coordination and support from development partners.

1. What efforts are being employed for peer-to-peer/web-based cervical cancer patient support including for marginalized or vulnerable populations?

- There were no significant efforts employed by the MOH to support peer-to-peer/web-based services including marginalized or vulnerable populations. Regarding peer-to-peer cervical cancer patient support, the MOH could draw lessons from the national HIV/AIDS program and implement them at national and regional levels in coordination with development partners and the private sector. All eligible women (age 30-49) who were visiting health facilities for various reasons such as MCH, FP, medical OPD, and ART clinics required to be considered for opportunistic cervical cancer screening services. Population-based screening could be helpful to reach a better number of eligible women nationally though it was found to be resource-intensive and require a correct central statistics agency (CSA) census report on targets. Messages from pre-cancer lesion-treated women and those dead from cervical cancer could be used to raise public awareness of cervical cancer prevention and control measures on different occasions.

1. **Creating the right incentive systems**

The following questions explore current incentive arrangements, how they influence the behavior of the actors in the system, and what impact this may have on the provision or consumption of cervical cancer services.

1. What are the payment rewards to providers for achieving or surpassing the required quality of care? Probe for any performance-based payment (PBP): a fee per beneficiary per month or monthly enhanced cervical cancer care.

- There were no incentive arrangements put in place to influence the behavior of providers such as a fee per beneficiary per month or payment for a monthly enhanced cervical cancer care for achieving or surpassing the required quality of care. This was not encouraged by the MoH due to its sustainability issues. It was considered to be one of the public healthcare services.

1. What are the mechanisms in place to support patients’ adherence to the prescribed medication, peer-to-peer support, transportation cost, or housing to ensure equitable access to facilities?

- There were no mechanisms put in place to support patients’ adherence to the prescribed medication, peer-to-peer support, transportation cost, or housing to ensure equitable access to facilities.

1. What are the major challenges faced by decision-makers while trying to implement the right incentive systems for providers and/or patients?

- The right incentive systems for providers and/or patients were not planned to be implemented by the decision makers at the national level as this was resource intensive and could result in disturbance of the whole healthcare system in the country.
