# Supplementary material for: Barriers and facilitators to implementation of the Ethiopian national cancer control plan strategies: Implications for cervical cancer services in Ethiopia
Source: PLOS Glob Public Health. 2024 Jul 22;4(7):e0003500. doi: 10.1371/journal.pgph.0003500 (PMC11262691; doi:10.1371/journal.pgph.0003500)
Supplement: S3 File — (ZIP) [file pgph.0003500.s003.zip › National Cancer Control Plan Data/10. KII List.docx]

KII 1 - MOH - Cervical cancer expert

KII 2 - MOH (EPI Director)

KII 3 - MOH (EPI officer)

KII 4 - MOH (EPI officer)

KII 5 - MOH (EPSS) – Director

KII 6 - MOH (EPSS) – Director

KII 7 - MOH (EFDA) – Director

KII 8 - MOH (EFDA) – Director

KII 9 - AACAHB – Cervical cancer expert

KII 10 - AACAHB (MCH Director)

KII 11 - AHF - PHS

KII 12 - CDC - Director

KII 13 - Pathfinder International - PHS

KII 14 - Wings of Healing - Director

KII 15 - FGAE - PHS

**Key:**

AACAHB: Addis Ababa City Administration Health Bureau; AHF: AIDS Healthcare Foundation; CDC: Centers for Disease Control and Prevention; EFDA: Ethiopian Food and Drug Administration; EPI: Expanded Program on Immunization; EPSS: Ethiopian Pharmaceuticals Supply Service; FGAE: Family Guidance Association of Ethiopia; KII: Key informant interview; MCH: Maternal and child health; MOH: Ministry of Health; PHS: Public health specialist

**N.B.** Eight directors, two cervical cancer experts, two EPI officers, and three public health specialists were involved in the key informant interviews.
