# Supplementary material for: Barriers and facilitators to implementation of the Ethiopian national cancer control plan strategies: Implications for cervical cancer services in Ethiopia
Source: PLOS Glob Public Health. 2024 Jul 22;4(7):e0003500. doi: 10.1371/journal.pgph.0003500 (PMC11262691; doi:10.1371/journal.pgph.0003500)
Supplement: S3 File — (ZIP) [file pgph.0003500.s003.zip › National Cancer Control Plan Data/11.TORs of the Cx Ca TWG.docx]

**Terms of Reference (TOR) of Cervical Cancer (Cx Ca) Technical Working Group (TWG)**

Under the leadership of the MOH NCD Case Team, the roles and responsibilities of the TWG include:

- Assisting in the overall joint planning of the national cervical cancer prevention (CCP) activities, ensuring that planned activities are within the national priority areas.
- Assisting in setting national targets for CCP based on national evidence and by adopting the WHO WHO’s 90-70-90 target to eliminate cervical cancer in Ethiopia by 2030.
- Ensuring the continuous provision of CCP services at the national level.
- Ensuring the establishment of a functional equipment maintenance system including on-time replacement of accessories for medical equipment used in the provision of cervical cancer service.
- Assisting in the introduction of new technologies and in closely monitoring their implementation as well as the outcome.
- Ensuring the establishment of a functional equipment maintenance system for medical equipment used in the provision of cervical cancer services.
- Ensuring availability and proper utilization of supplies for CCP activities.
- Conducting and coordinating. monitoring and evaluation, including the flow of health information.
- Assisting in strengthening and building partnerships and coordination for efficient utilization of recourses.
- Supporting the establishment of a center of excellence site for the provision of CCP training.
- Participating and also overseeing the quality of TOT training provided at the national and basic training provided at the regional level.
- Facilitating and conducting in-service training of health workers and program managers.
- Conducting regular program-focused supportive supervision to maintain the quality of services.
- Ensuring the provision of mentorship on CCP activities using regional health bureau (RHB) mentorship platforms.
- Assisting in the preparation of IEC/BCC materials and media messages, which aim to raise awareness of the general population as well as priority groups on cervical cancer prevention and control.
- Jointly develop-well-designed CCP advocacy, communication, and social mobilization/ IEC activities to ensure coherence of messages and proper target orientation being available and prepared to participate in meetings.
- Preparing national documents on CCP by adapting/adopting international recommendations to the Ethiopian context.
- Sharing information that is relevant to the group’s mandate, through meetings, interviews, public forums, or in writing.
- Participating in discussions before the group prepares its report.
- Collaborate with other activities under the NCD directorate or Maternal and Child Health (MCH) if there is an area of joint concerns (like HPV vaccination, palliative care, etc.), and
- Actively participate in the execution of specific tasks assigned to the group.
