# Supplementary material for: Barriers and facilitators to implementation of the Ethiopian national cancer control plan strategies: Implications for cervical cancer services in Ethiopia
Source: PLOS Glob Public Health. 2024 Jul 22;4(7):e0003500. doi: 10.1371/journal.pgph.0003500 (PMC11262691; doi:10.1371/journal.pgph.0003500)
Supplement: S3 File — (ZIP) [file pgph.0003500.s003.zip › National Cancer Control Plan Data/12. Thematic areas of the NCCP.docx]

Table. Thematic areas of the strategies supporting the national cancer control plan.

| # | Themes | Sub-themes |
| --- | --- | --- |
| 1 | Developing a political commitment | The commitment of the government |
|  |  | Stakeholders’ attention |
|  |  | Budget allocation |
| 2 | Creating transparent priority-setting | Setting priorities |
|  |  | Funding shortages |
| 3 | Strengthening interagency cooperation | Involvement in the technical working group |
|  |  | Multisectoral assistance |
| 4 | Integrating evidence into practice | Capacity of experts |
|  |  | Developing and disseminating guidelines |
|  |  | Training and monitoring of providers |
| 5 | Enhancing population empowerment | Programs for empowering communities |
|  |  | Patient support efforts |
| 6 | Creating the right incentive systems | Performance-based payment (PBP) |
|  |  | Patients’ support efforts |
|  |  | Decisionmakers challenges |
