# Supplementary material for: Barriers and facilitators to implementation of the Ethiopian national cancer control plan strategies: Implications for cervical cancer services in Ethiopia
Source: PLOS Glob Public Health. 2024 Jul 22;4(7):e0003500. doi: 10.1371/journal.pgph.0003500 (PMC11262691; doi:10.1371/journal.pgph.0003500)
Supplement: S3 File — (ZIP) [file pgph.0003500.s003.zip › National Cancer Control Plan Data/13. Summary of findings on strategies.docx]

**Political commitment**

The first lady with the minister of health was co-chairing the national cancer committee to guide cancer prevention and control activities. The Ministry of Health (MoH) deployed adequate personnel and allocation of funding for cervical cancer screening and treatment services in the country. Ethiopia developed the national cancer control plan (NCCP) in 2015 with a total cost of USD93,219, 270 covering 2015/16-2019/20. This was done in coordination with various development partners. Since the inception of cervical cancer prevention and screening in 2009, the Ministry of Health maintained progressive scale-up and equity of services in 1218 health facilities in 800 woredas (districts) of the country, with at least one health facility per district. The health facilities possess visual inspection with acetic acid and cryotherapy machines. Women mainly aged 30-49 were obtaining 100% free screening and treatment services at the public health facilities. However, this improvement did not result in public satisfaction especially due to inadequate access to and quality of diagnostic and treatment services at general and public hospitals in the capital. Cervical cancer annual review meetings were done with the involvement of regional and zonal focal persons for the evaluation of cervical cancer services. The MoH implemented combat cervical cancer (3Cs) service at cervical cancer screening and treatment facilities which aimed to screen 5 women in a day at a given health facility. The MoH provided mentorship of staff providing cervical cancer prevention and screening services in Addis Ababa. The MoH in partnership with development partners was involved in joint planning, capacity building, mentoring, technical support, financing, and provision of equipment and supplies. (Respondent in the disease prevention and control directorate, MoH, and NCDs unit, Addis Ababa City Administration Health Bureau)

**Priority setting**

Cervical cancer prevention and control were given priority by the government through coordination with various partners in the country. Improved community-based health insurance coverage, provision of diagnostic equipment, test kits, and reagents allowed the provision of free cervical cancer screening and treatment services at 1218 public healthcare facilities in the country. Income, place of residence, and ethnicity were taken into consideration when setting priorities by the government for cervical cancer services in the country.

**Primary prevention.** Though the HPV vaccine was required to be given as one of the routine immunization programs, in practice, all health facilities were doing it in campaigns. The national performance for July 2021 – June 2022 was 105% for HPV 1 and 84% for HPV 2 against 95% HPV 1 and 92% HPV2 of planned targets. On 24 September 2022, the state minister of health reported that the MoH in collaboration with the Ministry of Education the first dose of HPV vaccine was given to 4.8 million school girls; while the second dose was administered to 3.1 million school girls who turn age 14. Through the combat cervical cancer (3Cs) initiative, 367, 000 women were screened nationally from July 2021 to June 2022. A total of 900, 000 women were screened out of the eligible 10.7 million in the country equals 8.4%. It was far below the WHO’s 70% target for screening. In Addis Ababa, the cervical service achievement (plan versus performance) was HPV 1 vaccine (77%), HPV 2 vaccine (62%), screening (95%), and treatment (69%). The performance of HPV vaccination and treatment was way below the WHO 90-70-90 targets by 2030. The public awareness raising on cervical cancer in the community was found to be inadequate partly due to poor media coverage. An integrated refresher training on cervical cancer was not given in full to health extension workers (HEWs) as it was projected to cost over USD7.5 million. Therefore, the training was focused on urban settings but not on rural ones. (Respondents in the Expanded Program on Immunization (EPI), and disease prevention and control directorate in MoH)

**Secondary prevention**. The secondary prevention was focused on the see-and-treat approach with visual inspection using acetic acid (VIA) and treatment with cryotherapy and thermal ablation mainly for women aged 30-49 in 1218 health facilities, at least one screening center per woreda nationally. Pap smear test was not available in health facilities mainly due to the high cost and lack of infrastructure support. Clients were referred to the family guidance association (FGA) of Ethiopia or private diagnostic facilities which cost at least USD10 per test. The HPV DNA test was not regularly available in the facilities though its provision was supported by some of the partners in the country. The population-based screening was not implemented to reach a better number of eligible women nationally though it was found to be resource-intensive and required a correct central statistics agency (CSA) census report on targets. (Respondents in the disease prevention and control directorate in MoH, and partners)

**Tertiary prevention.** It was aiming to increase the number of tertiary-level hospitals providing chemotherapy, radiotherapy, and radical surgery services. In response to this, the Ministry of Health (MoH) established a few cervical cancer screening and treatment centers of excellence in various regions. The MoH reported that LEEP was done at 50 hospitals in the country while in Addis Ababa, seven hospitals were providing LEEP service. During this study, radiotherapy service was given at Tikur Anbessa, Jimma, and Haramaya specialized hospitals only, and the median waiting time was 7 months. Cervical cancer patients faced challenges to get treatment from specialized hospitals, and the median waiting time for chemotherapy service was 2 months. Lack of access to chemotherapy, radiotherapy, and radical surgery was a persistent issue, especially for patients referred to specialty care at specialized hospitals. Furthermore, the country lacked a population-based cancer registry in the country which did not allow for obtaining periodic and comprehensive epidemiolocal data on the most prevalent cancers nationally and implementing cost-effective strategies considering the available resources in the country. There was no comprehensive cancer surveillance system in the country. The population-based screening was not implemented to reach a better number of eligible women nationally though it was found to be resource-intensive and required a correct central statistics agency (CSA) census report on targets. There was no easily accessible system for the portability of patient records and information to specialized care and back to primary care that facilitates coordination of care across primary, secondary, and tertiary healthcare levels in Addis Ababa. Consequently, there was a lack of referral feedback for cervical cancer patients referred from primary care to specialized hospitals. (Respondents in the disease prevention and control directorate in MoH, and NCDs unit, Addis Ababa City Administration Health Bureau, and partners)

**Interagency cooperation**

The Ethiopian health policy requires integrated service delivery of the prevention and treatment of reproductive organ cancers implemented in partnership with various directorates of the Ministry of Health, regional health bureaus, and national and international development partners. Accordingly, the government of Ethiopia involved key stakeholders to mobilize resources in the fight against cancer. The Ministry of Health intensively facilitated cross-sectoral efforts to scale up cervical cancer prevention and control countrywide with the Ministry of Education, the Ministry of Women, children and youth affairs, and development partners in the country. The technical working group (TWG) was established for cervical cancer prevention and control including the Ministry of Health (MoH), hospitals, universities, partners, and others. The TWG was multisectoral and meets every 3 months. The TWG i) develops and revises guidelines and strategies including cervical cancer prevention and control guidelines, and mentorship guidelines; ii) adopts guidelines; iii) program implementation and monitoring; and iv) works on awareness of women in different sectors. The partners were working with the MoH in supporting health facilities, deployment of staff, technical support, capacity building, mentorship, supportive supervision, public awareness, financial assistance, HPV vaccine supply, provision of equipment, tests, and supplies. The quality of cervical cancer services was monitored by the MoH, regional, and zonal health offices with partner support at 200 health facilities in the country using the mentorship guidelines. The strategy for mobilization of pooled funding or joint budgeting and planning for the implementation of health in all policies (HiAP) from other government ministries and private sectors was not fully explored in Ethiopia. Multiple stakeholders including the private sector, media, and civil society were not adequately involved in the fight against cervical cancer in the country. (Respondents in the disease prevention and control directorate in MoH, NCDs unit, Addis Ababa City Administration Health Bureau, and partners)

**Integrating evidence into practice**

The first guideline for cervical cancer prevention and control in Ethiopia was launched in 2015 by the Ministry of Health (MoH) and revised in April 2021. The revised version provides the most current guidelines on public awareness, HPV immunization, risk identification, screening, early detection, diagnosis and treatment of cervical cancer at primary, secondary, and tertiary levels in Ethiopia. There was a national capacity for the development of cervical cancer prevention and control guidelines. The cervical cancer technical working group (TWG) was responsible to develop and review the cervical cancer guidelines. The members brainstormed, reviewed, and incorporated the global evidence and practice into guidelines, developed and reviewed training materials, cervical cancer prevention and control guidelines, cervical cancer mentorship guidelines, M & E tools, and information education and communication (IEC), and Social and Behavior Change Communication (SBCC) materials. The guidelines were aligned with the strategies of the national cancer control plan and followed a structured process under the leadership of the Ministry of Health (MoH). The MoH TOT training was provided to regional oncologists using guidelines, and the regions cascaded the basic training on identified gaps to health facilities. The MoH, regions, and zonal health offices managed mentorship and supportive supervision to monitor the implementation of guidelines during monthly, quarterly, and annual meetings. The guidelines were not incorporated into health professionals’ formal education. The regions participated in launching and disseminating the guidelines, supervision, mentoring, and training. (Respondents in the disease prevention and control directorate in MoH, and NCDs unit, Addis Ababa City Administration Health Bureau)

**Population empowerment**

With the support from MoH and its partners, some national radio and television messages were transmitted on cervical cancer prevention and control services for 3 months, 4 times per week. It was limited by the lack of resources. The study showed that health facility information solutions that enable patients to take a greater role in managing their health were limited to one-on-one and/or group education. There was no peer-to-peer or web-based cervical cancer patient support including for marginalized or vulnerable populations. Free hotline, mHealth, and eHealth on cervical cancer issues were not instituted to meet the demands of citizens. There were no influential and charismatic leaders or celebrities involved in the dissemination of cervical cancer prevention and control messages in the country. Messages from pre-cancer lesions-treated women were not considered to raise public awareness of cervical cancer prevention and control measures on different occasions. In Addis Ababa, there was no budget for raising public awareness through FM radio and Addis Media Network channels but some support was received from development partners. Population empowerment activities were not properly planned, coordinated, and adequately supported by development partners. (Respondents in the disease prevention and control directorate in MoH, and NCDs unit, Addis Ababa City Administration Health Bureau)

**Incentive systems**

There were no incentive arrangements put in place to influence the behavior of providers such as a fee per beneficiary per month or payment for monthly enhanced cervical cancer care, achieving or surpassing the required quality of care. There were no mechanisms put in place to support patients’ adherence to the prescribed medication, peer-to-peer support, transportation cost, or housing to ensure equitable access to facilities. The right incentive systems for providers and/or patients were not encouraged and planned to be implemented by the decision-makers at the national level as this was resource intensive and lacked sustainability. It was considered to be one of the public healthcare services. (Respondent in the disease prevention and control directorate, MoH)

**The national cancer control plan linkage with other policies and strategies**

The national cancer control plan (NCCP) recognizes the need for multi-sectoral involvement in the fight against cancer. It recommends comprehensive strategies to prevent and control cervical cancer through cross-cutting cervical cancer interventions and concerted efforts. Our documents review revealed that the Ministry of Health (MoH) put forward crosscutting cervical cancer interventions within the 1) National Health Sector Transformation Plan II (2020-2025) that targets to increase the proportion of women 30-49 years screened for cervical cancer from 5% to 40% (FMoH, 2021) with the public-private partnership in the country, 2) National Strategic Action Plan (NSAP) for control of non-communicable diseases (NCDs) recognizes that NCDs share similar risk factors and strategic prevention interventions including reduce exposure to tobacco use, harmful use of alcohol, and environmental carcinogens for cancer, 3) Guideline for Cervical Cancer Prevention and Control like the NCCP it has incorporated the public awareness, HPV immunization, risk identification, screening, early detection, diagnosis and treatment of cervical cancer at primary, secondary, and tertiary healthcare levels, 4) National Reproductive Health Strategy concurs with the NCCP and cervical cancer guidelines as sexually transmitted infection (STI) strategies address HPV through the implementation of nationwide vaccination programs by primary healthcare facilities, and 5) A Roadmap for Optimizing the Ethiopian Health Extension Program links with the NCCP through the health extension program (HEP) roadmap that requires health extension workers (HEWs) to disseminate public awareness messages, identify the most at-risk women for cervical precancerous lesions, and forward them to their respective public health centers.
