# Supplementary material for: Barriers and facilitators to implementation of the Ethiopian national cancer control plan strategies: Implications for cervical cancer services in Ethiopia
Source: PLOS Glob Public Health. 2024 Jul 22;4(7):e0003500. doi: 10.1371/journal.pgph.0003500 (PMC11262691; doi:10.1371/journal.pgph.0003500)
Supplement: S3 File — (ZIP) [file pgph.0003500.s003.zip › National Cancer Control Plan Data/2. HPV Vaccine_EPI_MOH.docx]

**Program and Supply Management of Human Papillomavirus (HPV) Vaccine**

1. **When was the HPV vaccine first implemented in Ethiopia?**

HPV vaccination was introduced in December 2018 after a two-year demonstration in 2 districts of the Oromia and Tigray regions.

1. **Who is responsible for the quantification of the HPV vaccine?**

The HPV quantification was done based on the number of targets and expected coverage. The quantity was determined by GAVI since it was a donation and renewal done with other RI vaccines.

1. **What are the quantification methodologies, data, and tools in terms of:**
2. **period covered:** It was annually based
3. **stakeholders involved:** MOH with UNICEF and GAVI
4. **method: Based:** On the target and expected coverage
5. **key assumptions/targets:** Age groups (14) both at school and out of school
6. **source of data:** Headcount and conversion factor from the general population
7. **tools used:** There was a micro-planning template to do planning for the vaccination campaign
8. **reference unit price:** The price was calculated /determined from the UNICEF Supply catalog
9. **commodity gap:** no response
10. **Who manages the procurement of the HPV vaccine?**

- UNICEF was in charge of the procurement of HPV and all vaccine supplies in the country**.**

**How long are the procurement lead times?**

- The procurement was managed by the GAVI and UNICEF supply division and there was a definite delivery schedule.

1. **What are the in-country distribution mechanisms in place?**

- The distribution plan was prepared by the MoH and shared with central EPSS.
- Based on the MoH distribution plan: vaccines, supplies, and printed materials were distributed to EPSS hubs.
- EPSS hubs then distributed HPV vaccines, supplies, and printed materials to woredas, and finally, woredas were distributing the commodities to HFs.

1. **What are the reporting mechanisms from a health facility to a woreda, zone, region, and ministry of health?**

- The performance reports are collected through reporting template manually and through DHIS2.

1. **What are the areas of coordination with and involvement of stakeholders (Gavi, development partners, NGOs, international organizations, donors)?**
   1. Gavi covered the operational cost during the demo project and national rollout. The vaccine cost was also covered by GAVI. Technical, financial, and logistical support was also obtained from different in-country partners including WHO, UNICEF, CHAI, PATH, Girl Effect, PSI, and Jhpiego.
   2. There were multiple coordination meetings with partners during the vaccination campaign.
   3. Communication, logistics, and service delivery. TWG had frequent meetings during the campaign.
   4. Virtual orientation for RHBs, national and sub–national advocacy meetings.
   5. Agencies including EPSS, EFDA, and EPHI were also engaged in the TWG.
2. **What is the rate of national performance versus the plan in the last 12 months?**

- The plan for HPV 1 and 2 in the last 12 months was 95% and 92% respectively. The performance showed 105% for HPV 1 and 84% for HPV 2.

1. **What are the challenges or gaps encountered with the program and supply chain management of the HPV vaccine and how they have been tackled?**
   1. Poor stock management of leftover vaccine.
   2. Poor bundling: missing items during distribution.
   3. Gaps in cold chain management.
   4. Transportation challenge for timely distribution.
   5. Program interruption due to the COVID-19 pandemic resulted in the drop-out of target girls.
   6. Lack of operational cost.
   7. Age trade-off (under and over age vaccination).
2. **Is there any new implementation strategy?**

- Vaccine delivery modalities were mainly school-based and in a campaign mode. In addition, HF, outreach, and mobile teams were used based on the context at the ground. With regards to dosage, the country follows two-dose administration.

1. **Other points**

- The quantification of the HPV vaccine was done by the MOH, Gavi funds the procurement and procured by Unicef. The Gardasil vaccines (quadrivalent) were supplied by MSD. The vaccine was introduced in December 2018.
- On 24 September 2022, the state minister of health reported that the MoH in collaboration with the ministry of education the first dose of HPV vaccine was given to 4.8 million school girls; while the second dose was administered to 3.1 million school girls who turn age 14.
- For HPV1 and HPV2 - Seven days vaccination campaign was conducted in Addis Ababa in January 2022 (for the reporting period July 2021-June 2022). It was not given in a routine vaccination program.
