# Supplementary material for: Barriers and facilitators to implementation of the Ethiopian national cancer control plan strategies: Implications for cervical cancer services in Ethiopia
Source: PLOS Glob Public Health. 2024 Jul 22;4(7):e0003500. doi: 10.1371/journal.pgph.0003500 (PMC11262691; doi:10.1371/journal.pgph.0003500)
Supplement: S3 File — (ZIP) [file pgph.0003500.s003.zip › National Cancer Control Plan Data/3. Ca Medicines_EPSS.docx]

**Ethiopian Pharmaceuticals Supply Service (EPSS) quantification and procurement of cancer medicines**

1. The quantification for cancer medicines considers an estimated number of cancer patients out of the projected number of patients expected to be seen in the gynecology clinic. The drug and supply management (DSM) team quantifies the medications accordingly. This is communicated to EPSS for procurement purposes. The number of patients to be seen become 12 times in the last 5 years.

2. The chemotherapy-providing general and specialized hospitals were selected by the MoH.

3. The quantities for cancer medicines procurement were based on the annual demand of service to be provided by the general or specialized hospitals.

4. The quantification was done using consumption and morbidity methods. These were considered for triangulation of the quantification outcomes.

5. Hospitals quantify their needs based on consumption and morbidity methods. They also consider cancer medicines prescribed and procured from outside by patients. The facilities attend the analysis and validation workshop which was conducted for 2 days by MoH, EPSS, EFDA, and other stakeholders.

6. Budget for the procurement of cancer medicines- 50% of the budget was fully subsided from the MoH based on demand and 50% from the respective hospitals. There was no direct budget transfer by the hospitals but expected to pay after the delivery of products.

7. Supply plan was done after the completion of the quantification.

8. Challenges:

- There was no cancer medicines STGs
- There was no national cancer registry.
- Registration with EFDA was mandatory for the procurement of cancer medicines.
- Disposal of expired or damaged cancer medicines was a challenge as these were recommended to be returned to the suppliers.
- Direct procurement of small quantity cancer medicines required approval by the PPDA.
