# Supplementary material for: Barriers and facilitators to implementation of the Ethiopian national cancer control plan strategies: Implications for cervical cancer services in Ethiopia
Source: PLOS Glob Public Health. 2024 Jul 22;4(7):e0003500. doi: 10.1371/journal.pgph.0003500 (PMC11262691; doi:10.1371/journal.pgph.0003500)
Supplement: S3 File — (ZIP) [file pgph.0003500.s003.zip › National Cancer Control Plan Data/4. Ca Medicines Regulation_EFDA.docx]

**Regulation of cancer medicines by the Ethiopian Food and Drug Administration (EFDA)**

**Registration and marketing authorization**

- EFDA focuses on food, medicines, and medical devices. Reviews and approves products applied for registration by manufacturers, importers, and wholesalers. It also follows or monitors products after marketing authorization are allowed.
- An individual, importer, and consultant are allowed to register medicines, equipment, and medical devices. However, a license to run the business is required during the importation of products. There is no limit for manufacturers’/suppliers’ agents in the country.
- Cancer medicines are registered with a fast-track system.
- It considers stringent regulatory authority (SRA) abbreviated review. So, GMP is a waiver for countries including Japan, Australia, the USA, Canada, the UK, European Union… If SRA approves marketing authorization or sales of a product from other countries in their country, it is accepted/approved by EFDA.
- The registration of WHO-prequalified products could take up to 90 days.
- Outside SRA or WHO prequalification, the GMP inspection is done by EFDA. The GMP application and inspection process for this takes from 3-6 months. The application and inspection are done in parallel. This has shortened the time required for the registration of products.
- WHO collaborative registration procedure is done by WHO and EFDA regulatory experts. Documents are shared between WHO and EFDA. In case there is a need for face-to-face discussion, the EFDA experts travel to WHO offices in Geneva.
- To achieve price competition EFDA allowed multiple agents for the importation of products tax-free. The review and approval of cancer medicines are done with a fast-track system. PPP is open to any interested body in the country.

**Inspection of cancer medicines**

- There could be a possibility for the infiltration of unregistered cancer medicines into the country. There was a high demand for cancer medicines mainly due to the burden of non-communicable diseases (NCDs) is increasing because of the growth and aging of the population, as well as an increasing prevalence of established risk factors, such as smoking, overweight, physical inactivity, and changing reproductive patterns associated with urbanization and economic development (FMoH, 2015). Moreover, Ethiopia is bounded by fragile and insecure countries including Somalia and South Sudan. People could also carry them using their bags through airports. During this study, there was no report on the prevalence of substandard/counterfeit cancer medicines. EFDA was doing a quantitative survey in collaboration with Addis Ababa University, School of Pharmacy (November 2021 - 2022). The legal and illegal products will be checked against port clearance data. The products will be collected from pharmacy outlets and planned to be tested using Minilabs. The report is expected in the coming two months.

**Guideline for conditional approval of medicines. (Dec. 2020).** **ETHIOPIA FOOD AND DRUG AUTHORITY (EFDA).**

The Ethiopian Food and Drug Authority (EFDA) is mandated, in Proclamation 661/2009, to ensure the safety, quality, and efficacy of medicines. To achieve this, the authority has been working on different regulatory activities.

- **Inspection:** good manufacturing practice inspection and port inspection;
- **Evaluation:** standardized premarketing dossier evaluation;
- **Analysis:** quality control analysis;
- **Monitoring:** post-marketing quality and safety monitoring of the medicines.

The **medicine market authorization** is one of the top priority areas of EFDA. In addition to the dedicated assessors, the authority uses a national drug advisory committee for the assessment and registration of medicines. While an alternative marketing approval pathway is devised to provide access to certain medicines for the unmet medical need of the public such as medicines for seriously debilitating diseases or life treating diseases such as cancer.

1. **Eligibility Criteria**

**The following criteria should be fulfilled for conditional approval**

1. The benefit–risk balance of medicine is positive.
2. The applicant will likely provide comprehensive data.
3. Unmet medical needs will be fulfilled.
4. The immediate availability of the medicine on the market outweighs the risks due to the need for additional data.
5. An early access pathway for medicines that show promising therapeutic effects but for which comprehensive data are not available.
6. Investigational medicinal products are under clinical trial phase 3 but are required for critical life-threatening diseases which have no other alternatives.
7. **Technical and Administrative Requirements**

The applicant must submit/provide at least the following information and/or documents:

- 1. Cover letter
  2. Filled and signed the application form as per the guideline for registration of medicine of the authority
  3. Summary of available data that shows the risk-benefit balance of the product is positive
  4. Product dossier in CTD format including the available protocol for those data not submitted.
  5. Proof that the conditional approval will fulfill the unmet medical needs
  6. A commitment letter from the applicant should be submitted to provide comprehensive data as per the proposed time frame indicated by the applicant.
  7. Commitment letter to submit the Authority periodic safety update report every six months following granting and/or renewal of approval with conditional approval pathway.
  8. Evidence that shows a medicine has been granted conditional approval by WHO or SRA, if available; and
  9. Documents listed below:
- Agency Agreement.
- Good Manufacturing Practice- A copy of valid current good manufacturing practice (cGMP) certificate or GMP waiver letter issued by EFDA. A copy of valid current good manufacturing practice (cGMP) certificate issued by a national authority in the country of origin or SRA.
- Product information, labeling, and patient information leaflet (PIL) or package insert.
- Evidence for payment of service fees.

1. **PERIOD OF APPROVAL: - *The maximum period for provisional marketing authorization of medicines under conditional approval is limited to a maximum of one year.***
2. **Review Process: -** When the application is submitted to the authority, the director and/or team leader appoints a team of experts for review. Experts review the application and provide a summary report and recommendations to the director and/or team leader of the Medicine Registration and Licensing Directorate of the Authority.

**What could be considered by EFDA to ensure better availability and affordability of cancer medicines in the country?**

a. Provide price information- EFDA provided open access to the electronic registration information system (eRIS) but not prices.

b. Ensure price competition- EFDA does this through the registration of an unlimited number of suppliers.

c. Apply price control measures- this was not EFDA’s mandate

d. Reduce duties/taxes/markups- cancer medicines were treated like any other pharmaceuticals.
