# Supplementary material for: Barriers and facilitators to implementation of the Ethiopian national cancer control plan strategies: Implications for cervical cancer services in Ethiopia
Source: PLOS Glob Public Health. 2024 Jul 22;4(7):e0003500. doi: 10.1371/journal.pgph.0003500 (PMC11262691; doi:10.1371/journal.pgph.0003500)
Supplement: S3 File — (ZIP) [file pgph.0003500.s003.zip › National Cancer Control Plan Data/5. AACAHB_transcripts.docx]

**Appendix 3: Qualitative Research Instruments**

- 1. **Exploring Factors Affecting the National Cancer Control Plan Implementation.**

**Objective 1:** To examine the extent to which the Ministry of Health's existing institutional strategies for improving access to and quality of cervical cancer services support the national cancer control plan implementation framework for Ethiopia.

**INTERVIEWER:** Good morning/afternoon. Introduce yourself and ask the key informants to introduce themselves. Ensure that the consent form is signed by individual participants. Then explain the following questions that will explore the national cancer control plan implementation challenges to access quality cervical cancer services.

1. **Developing a political commitment to quality cervical cancer prevention and control**

The following questions aim to understand the level of political commitment by the national/regional government to improve cervical cancer care.

1. What is your opinion regarding political commitment by the government/national political leadership for cervical cancer care in the country?

- The political commitment was excellent. All health facilities possess Cryotherapy machines and an adequate number of health workers were trained in cervical cancer (Cx Ca) prevention, screening, and treatment services.

2. What do you think about the level of attention given to cervical cancer by activists, cancer societies, citizens, civil society organizations, intellectuals, and patients during the FMoH annual review meeting (ARM) or annual NCDs meeting?

- The Ministry of Health invites all regions to the annual meeting on non-communicable diseases (NCDs) yearly and specific for Cx Ca was conducted in 2021/2022. The annual meetings focused on HPV vaccination, screening, treatment, and challenges encountered in the regions. The weekly pre-cervical cancer lesions screening and treatment reports were sent by the health facilities to the AACAHB. Moreover, mentorship services were provided for 20 public health centers for 20 days (1 day per facility) by MoH in coordination with AACAHB. This was done by 2 professionals from hospitals (who took TOT and mentorship orientation by MOH) and 1 health officer (Cx Ca focal person) from the AACAHB. The mentorship in these health centers was provided to health professionals who received Cx Ca TOT training and to those providing Cx Ca prevention and screening services. Their skills were assessed and following this feedback was given on the spot and a written report was provided to the health facility managers.

3. What efforts have been made to increase public funding during the health budget allocation process considering the cost-effectiveness of cervical cancer prevention and control services?

- There was no constant budget set for NCDs and Cx Ca services in AACAHB. The majority of the cervical cancer budget was allocated by the Ministry of Health (MOH) and its partners work in coordination with MoH and regional health bureaus to allocate resources and fill the gaps in the provision of Cx Ca prevention and control services in terms of training, financing, population empowerment, and provision of equipment and supplies.

1. **Creating transparent and evidence-based priority-setting**

The following questions are intended to identify whether transparent procedures and evidence-based priority-setting approaches exist.

1. What are the measures being taken into account when setting priorities by the government on cervical cancer services? Probe for equity (income, place of residence, and ethnicity).

- There were no issues of equity. HWs trained, equipment available, and service provided at no cost (for screening and treatment). The issues are when clients were referred for chemotherapy, radiotherapy, and radical surgery.

1. What are the essential cancer medicines, diagnostic tests, or equipment that health facilities would like to have but cannot get funding for?

- Already has been addressed above.

1. What happens to those who do not receive cervical cancer care for certain conditions because of limitations in public funding?

- There was nothing to be done in this area due to limitations in public funding.

1. **Strengthening interagency cooperation**

The following questions focus on documenting mechanisms for interagency cooperation and their effectiveness.

1. What are the terms of reference (TOR) of the national cancer committee (NCC)? How effective is the NCC's involvement in goal and target-setting, policy implementation, monitoring, and reporting on cervical CA care in the country? Inquire for the TOR.

- The region has NCDs TWGs with the involvement of partners but not specific for Cx Ca – Wings of Healing (works on Cx Ca) and others working on hypertension, DM, and COPD.

1. What specific steps have been taken by the FMOH to mobilize multisectoral assistance from other sectors for the implementation of health in all policies (HiAP) and/or pooled funding?

- The NCDs TWGs meetings were conducted quarterly including the Cx Ca issues- the performance and challenges on screening and treatment were presented and discussed at the meeting.
- The gender directorate of AACAHB – works on the dissemination of public awareness such as on 8^th^ March. Family health team (comprises of 8-10 personnel)- teach women the Cx Ca prevention and screening during their outreach visits.
- There was some liaison with AACAEB. However, this area was not adequately exploited and it needs to be strengthened, i.e., with AACAEB, women, youth, and child affairs, and sports bureau, and others.

1. **Integrating evidence into practice**

The following questions explore mechanisms to integrate evidence into medical practice.

1. What is your opinion on the capacity of the unit responsible to develop and review cervical cancer guidelines?

- There was adequate capacity at the national level.

1. What is the process for developing and disseminating cervical cancer clinical guidelines, providers training, and monitoring whether providers adhere to guidelines?

- AACAHB participates in the launching and dissemination of the guidelines.
- Supervisions and mentoring were done by AACAHB.
- The training was organized by the MOH in coordination with AACAHB (sends professionals for training) and partners (provide funding and training).
- WHO (NORAD) provides funds and works with Bole and NSL sub-cities in a few health facilities.
- FGA provides training or it was done by hospitals accredited for providing CPD training.

1. How-if at all-are new guidelines incorporated into health professionals’ education and/or continuing education? In practice, who provides the training?

- The MOH training was conducted using Cx Ca guidelines but not incorporated into health professionals’ formal education.

1. **Enhancing population empowerment**

The following questions explore mechanisms and efforts to empower people to be frontline workers for cervical cancer care.

1. What policies, programs, or guidelines are designed to empower communities and service users to take responsibility for identifying their own needs and preferences, managing their health with appropriate support from health-service providers, and understanding where cervical cancer services are available?

- The policy for enhancing population empowerment was developed by MOH and implemented by AACAHB. There was no budget set by the MOH but some support was received from CDC. Apart from this, the communication director in the health bureau liaises with other partners for any support. Moreover, it was done whenever the government media approaches the AACAHB for disseminating any public awareness messages.

1. What efforts are being employed to peer-to-peer/web-based cervical cancer patients support including for marginalized or vulnerable populations?

- There were no peer-to-peer/web-based cervical cancer patients support including for marginalized or vulnerable populations implemented by the bureau. Moreover, the cervical cancer survivors were not engaged to share their experiences with cervical cancer screening and treatment opportunities and challenges. The region is expected to engage cervical cancer living witnesses to improve the uptake and utilization of screening and treatment of cervical cancer.

1. **Creating the right incentive systems**

The following questions explore current incentive arrangements, how they influence the behavior of the actors in the system, and what impact this may have on the provision or consumption of cervical cancer services.

1. What are the payment rewards to providers for achieving or surpassing the required quality of care? Probe for any performance-based payment (PBP): a fee per beneficiary per month or monthly enhanced cervical cancer care.

- There were no incentives put in place.

1. What are the mechanisms in place to support patients’ adherence to the prescribed medication, peer-to-peer support, transportation cost, or housing to ensure equitable access to facilities?

- None

1. What are the major challenges faced by decision-makers while trying to implement the right incentive systems for providers and/or patients?

• Resources constraint and sustainability issues.
