# Supplementary material for: Barriers and facilitators to implementation of the Ethiopian national cancer control plan strategies: Implications for cervical cancer services in Ethiopia
Source: PLOS Glob Public Health. 2024 Jul 22;4(7):e0003500. doi: 10.1371/journal.pgph.0003500 (PMC11262691; doi:10.1371/journal.pgph.0003500)
Supplement: S3 File — (ZIP) [file pgph.0003500.s003.zip › National Cancer Control Plan Data/6. AHF activities.docx]

**AIDS Healthcare Foundation cervical cancer activities**

1. What type of partnership does your organization have with the MOH and/or AACAHB and/or health facilities to foster excellence in cervical cancer prevention, screening, early detection, diagnosis, and treatment?

Response:

AIDS Healthcare Foundation (AHF) has a partnership agreement, Memorandum of Understating (MOU), with the Federal Ministry of Health, the former Federal HIV/AIDS Prevention control office (HAPCO), and AACAHB. This partnership particularly with AACAHB encompasses the prevention and management of cervical cancer (CC) in supported health facilities. In AACAHB, AHF is supporting 4 health centers and two federal hospitals namely ALERT and St Paul’s Hospital Millennium Medical College.

1. What specific supports have been provided to the MoH, AACAHB, and health facilities in terms of:
2. human resource (capacity building/training/mentorship)
3. technical support
4. financial assistance
5. public awareness (audio/video/print media)
6. improving access to medicines/vaccines/medical supplies/equipment
7. strengthening the cervical cancer program and logistics information system

Response:

AHF is supporting the health facilities with all necessary supplies of materials and equipment, training, mentorship, and Provision of drugs and reagents required to screen and manage CC. AHF also arrange referral to a nearby hospital and private health facility for further diagnosis and management of CC whenever the client needed such service. This referral is free of charge and AHF covers the cost incurred during referral to the private facilities.

1. What do you think are the major health system factors (such as organizations, products, people, and actions) that impact the cervical cancer service delivery in Addis Ababa/Ethiopia?

Response:

Throughout our intervention we have observed that the following are major challenges:

1. The lack of motivation of the health provider to elicit and offer the service is more of a passive service and on demand of the client than specifically requested and offered for all eligible clients.
2. Unfriendly workflow of the health service: in most health facilities the units providing CC service are far, less suitable, and less friendly.
3. Shortage of input: Materials, equipment, reagent
4. Lack of awareness of the need for the CC screening
5. Mentorship and follow-up from higher government offices and facility management
6. Trained staff attrition and delay in replacing the vacant position
7. What health system components (such as human, physical, and financial) should be given attention, investment, and prioritization to achieve minimized direct cost, improved population coverage, and promoted service coverage of cervical cancer prevention and control in Addis Ababa/Ethiopia?

Response:

The intervention shall address the challenges listed above.

1. Maximum attention, and clear understanding of the magnitude and gravity of the problem, by all levels of the facility and service management
2. A team-based approach to the service provision
3. Creating a friendly workflow for the clients of the CC screening and management
4. Uninterrupted supply of reagents, materials equipment
5. Ensure free-of-charge CC screening and management at health facilities (initial and referral sites)
6. Demand creation among the community
7. Improved partner involvement and participation
8. Any other information that you think is important about your organization’s partnership with MoH/AACAHB regarding cervical cancer service delivery?

Response:

AHF advocate the wider service provision of CC screening and management and greater Government and international partner organization. CC account for major causes of morbidity and mostly in low-income countries and among the HIV-positive population. It is of high importance to engage the MOH and local partners to enact the policy and recommendation in the guidelines. Facility management and more importantly the health care providers play a pivotal role to provide quality, friendly, interrupted service for provider-initiated clients and those who are coming to health facilities through referrals by community health workers and health extension workers.

**Other activities**

- AHF supports were facility need-based.
- Built 3 rooms for cervical cancer screening and treatment services. It commenced its service in March 2022. There were no provider service interruptions afterward. It becomes a suitable working environment.
- There is a credit agreement with pharmacies for medications.
- Imaging contract with Wudassie Diagnostics (for MRI).
- CT is available in the St. Paul hospital but no contrast media. So, clients are sent outside for this diagnostic test.
- There is a Pap smear contract with Arsho Lab (30 tests per month).
- The reasons for low Pap smear tests i) tests were requested by AHF but clients did not show up at Arsho clinic for the tests, ii) tests were done but clients did not collect the results, iii) tests were done at Arsho clinic and the results received by clients but they did not show up for undisclosed reasons. However, AHF has started to receive the clients' results through email and call them in case they do not show up for follow-up services.
- LTFU clients were reached with phone calls (supported by AHF).
- Five (5) trained nurses can take Pap smear at the ART clinic. This can be forwarded to the Arsho clinic for testing. The samples could be collected by motorbikes. However, this was hampered by a shortage of supplies (brush, smear-making spatula…). There was a shortage of Pap smear supplies in the national market. So, Arsho Lab was forced to reuse the supplies.
- AHF provided up to 40 gowns to ART clinic staff.
- Disposable and surgical gloves were provided for the whole ART clinic services.
- Refresher courses were provided to various staff.
- AHF provided a FACSPresto™ machine- for CD4 baseline tests for newly enrolled patients (buffer, chemistry, CBC, VDRL…). Follow-up tests were done at ICL. While VL was done at Ethiopian Public Health Institute (EPHI).
