# Supplementary material for: Barriers and facilitators to implementation of the Ethiopian national cancer control plan strategies: Implications for cervical cancer services in Ethiopia
Source: PLOS Glob Public Health. 2024 Jul 22;4(7):e0003500. doi: 10.1371/journal.pgph.0003500 (PMC11262691; doi:10.1371/journal.pgph.0003500)
Supplement: S3 File — (ZIP) [file pgph.0003500.s003.zip › National Cancer Control Plan Data/7. CDC activities.docx]

**CDC cervical cancer activities**

1. **What type of partnership does your organization have with the MOH and/or****AACAHB and/or health facilities to foster excellence in cervical cancer prevention, screening, early detection, diagnosis, and treatment?**

The Centers for Disease Control and Prevention (CDC) works closely with MOH and HFs on cervical cancer prevention programs in various areas to reach eligible population groups. While working in partnership, all implementing actors are expected to play the following roles attached to them individually.

**Ministry of Health (MOH)**

- The overall policy formulation, strategy development and revision, development, and review of guidelines and standard operating procedures, program management and coordination, quality assurance, as well as ensuring institutionalization of the program into routine service delivery giving overall programmatic guidance is done by MOH.
- Capacity building of HCP through basic and TOT training on cervical cancer screening and treatment is cascaded by MOH to the region.
- The Ministry ensures uninterrupted disposal of supply to cervical cancer screening and treatment services including ensuring availability and local production of quality and standard acetic acid solutions. Different Machines, equipment, and M&E tools (Cryomachine, Acetic acid speculum guidelines, and registers) are provided to the regions.
- The Ministry produces local evidence through research and surveillance, adopts international scientific recommendations, and disseminates to regions through various methodologies for action.
- There are different face-to-face as well as virtual sessions between MOH and regions to monitor the overall program.
- MOH is in charge of the coordination of all the national activities and all stakeholders with an interest in cervical cancer prevention activities and ensuring linkages with other government sectors and non-governmental organizations. The RMNCH Directorate is responsible to undertake and sustain the routinization of HPV vaccination for eligible target girls.

**Addis Ababa City Administration Health Bureau (AACAHB)**

- Regional Health Bureau (RHB) adapt the technical guidelines from the Ministry to implement interventions on the prevention and control of cervical cancer and provision of screening (VIA or HPV testing) and treatment (cryotherapy/ thermal ablation/LEEP (Loop Electrosurgical Excision Procedure)) services in their regional context. The RHBs are in charge of planning, implementing, coordinating, monitoring, and evaluating the screening and treatment services in particular and prevention and control of other cervical cancer activities including routinization of HPV vaccination within the regions.
- RHB is also responsible for capacity building of HCP basic and refresher training on cervical cancer screening and treatment given to health facilities (HFs).
- The RHB ensures uninterrupted supply to cervical cancer screening and treatment including ensuring availability of quality and standard acetic acid solutions. Different equipment, supplies, and M&E tools (Acetic acid speculum guidelines, registers, and other PSTs) are provided to the HFs by the region.
- There are different face-to-face as well as virtual sessions between RHB and SCs as well as HF to monitor the overall program.
- The region also produces different spot media messages, and print and disseminates IEC/BCC materials to increase public awareness of cervical Ca.
- The RHB also gives technical assistance to SC and HFs through regular mentorship and supervision.

**Health facilities**

- Health facilities (hospitals and health centers) have a role in the demand creation and provision of VIA and cryotherapy/thermal ablation services for eligible women in their catchment areas. In addition, selected high-load hospitals provide LEEP service that covers other nearby health centers through referral networking. They ensure the Availability of commodities, supplies, and equipment to facilitate cervical cancer screening in their Specific Sites; and ensure that equipment is always in good condition (servicing and repairs as necessary), Follow-up of clients (especially screen-positive clients) to ensure that they complete treatment and adhere to management protocols, completion of the requisite data tools /registers and submission reports in a timely, accurate and consistent manner, community mobilization activities in their areas of operation and availability of IEC / BCC materials And technical support, outreach and in reach cervical cancer screening and treatment activities, administer HPV vaccination for eligible girls as per the national immunization schedule.

**2.    What specific supports have been provided to the AACAHB, and health facilities in terms of?**

**A.**  **Human resource (capacity building/training/mentorship)**

The following human resources are deployed to support cervical cancer prevention, screening, and management program implementation at different levels:

- One regional-level adviser, namely; Cervical cancer prevention, screening, and management adviser
- 11 Sub-city health office cervical cancer prevention, screening, and management coordinators at 11 SCHOs
- 47 care and treatment officers that also work as cervical cancer prevention, screening, and management officers at 47 HFs

Training is being given to the facility-level providers, sub-city and regional level coordinators, and advisers on different topics such as Basic and refresher training on Cx-Ca screening and treatment for HCPs including new initiatives (Thermal ablation), Basic LEEP training for gynecologists and HCPs, Managerial training on Cx-Ca screening and treatment for HCPs and training on HPV DNA Test for Health Care providers.

**Mentorship** as a technical support service is also provided to all implementing HFs by trained mentors at least monthly.

**B.**  **Technical support**

- Technical supports are expressed in terms of regular mentorship and supportive supervision which are given to all implementing HFs by sub-city and RHB staff.
- The guidelines, SOP, and other basic program M&E documents are also customized and distributed as part of TA.
- Any technical (programmatic and clinical) counselling requests are being addressed

**C.**  **Financial assistance**

- Financial support is given to the region to undertake different activities like capacity building sessions, program reviews, and supervisions, to avail program supplies, produce and disseminate awareness creation sessions, and to cover personnel cost of technical assistance at all levels

**D.** **Public awareness (audio/video/print media)**

- TV spot media messages are produced and disseminated through different channels to create public awareness, IEC/BCC materials are also printed and distributed to the public through HF

**E.  Improving access to medicines/vaccines/medical supplies/equipment**

- Equipment such as speculum, forceps, kidney dish, galipot, examination light, and supplies like Acetic acid, alcohol, iodine, glutaraldehyde, and sanitary pad are purchased and distributed to facilities to ensure sustainable service provision.

**F.**   **Strengthening the cervical cancer program and logistics information system**

- Cervical cancer program-related equipment and supplies are managed under the regional logistics information system within the regional supply chain management system. So any effort to strengthen LIS will also benefit the cervical cancer program-related equipment and supplies information management.

**3.**    **What do you think are the major health system factors (such as organizations, products, people, and actions) that impact the cervical cancer service delivery in Addis Ababa/Ethiopia?**

- Some of the factors impacting the program are having strong integration and communication between stakeholders, engagement of concerned leaders in monitoring the program implementation, continuous and sustainable service delivery at every facility, having strong supply chain management to ensure availability and readiness of the necessary equipment and supplies.
- Existing strong capacity-building schemes and technical assistance could result in the availability of trained and skilled manpower.
- Having a strong referral network system to strengthen service accessibility to the public and community awareness creation sessions that improved public awareness.

**4.**    **What health system components (such as human, physical, and financial) should be given attention, investment, and prioritization to achieve minimized direct cost, improved population coverage, and promoted service coverage of cervical cancer prevention and control in Addis Ababa/Ethiopia?**

- Awareness creation to the target population to let them seek screening services as early as possible and create optimal access to the service at the nearest possible service outlets including community posts must be emphasized.
- Our screening and treatment approaches must take cultural issues into consideration
- Importing/producing basic program equipment and supplies must be given due attention to have well-equipped health facilities that meet the standard.

**5.    Any other information that you think is important about your organization’s partnership with MOH/AACAHB regarding cervical cancer service delivery?**

- Domestic resource mobilization must be given attention to achieve program sustainability. Well-organized partner management is also important to properly utilize the available resources, avoid redundancies and cover actual program gaps.
