# Supplementary material for: Barriers and facilitators to implementation of the Ethiopian national cancer control plan strategies: Implications for cervical cancer services in Ethiopia
Source: PLOS Glob Public Health. 2024 Jul 22;4(7):e0003500. doi: 10.1371/journal.pgph.0003500 (PMC11262691; doi:10.1371/journal.pgph.0003500)
Supplement: S3 File — (ZIP) [file pgph.0003500.s003.zip › National Cancer Control Plan Data/8. WOH_Responses.docx]

**WINGS OF HEALING CERVICAL CANCER SCREENING ACTIVITIES**

1& 2. Wings of Healing (WH) partnership is mainly on secondary prevention of cervical cancer through screening of eligible women. Our project is comprehensive as we first create awareness about cervical cancer screening since there is a huge awareness gap.

We will then screen the eligible women, and treat all who tested positive on the same day and for those who need further management like chemotherapy/radiotherapy, our company will cover the cost. Apart from this WOH has been training health professionals on basic cervical cancer screening and treatment and working on health facility capacity building.

Summary of Wings of Healing activities

| Screening | 7000 women |
| --- | --- |
| Training | 47 health providers |
| Capacity building | Distributed 250 sponge forceps, 100 vaginal speculum, and 50 gallipots |
| Awareness creation | More than 10,000 women |

3. The major health system factors are the lack of proper awareness about cervical cancer screening in different public media.

4. Attention should be given to creating awareness and providing basic cervical cancer screening and treatment training for mid-level health professionals. We even suggest that basic cervical cancer screening training can be incorporated into the medical curriculum.

5. Our strong collaboration and the need-based, effective, innovative, and comprehensive approach have contributed a lot to our successful project.
