# Supplementary material for: Barriers and facilitators to implementation of the Ethiopian national cancer control plan strategies: Implications for cervical cancer services in Ethiopia
Source: PLOS Glob Public Health. 2024 Jul 22;4(7):e0003500. doi: 10.1371/journal.pgph.0003500 (PMC11262691; doi:10.1371/journal.pgph.0003500)
Supplement: S3 File — (ZIP) [file pgph.0003500.s003.zip › National Cancer Control Plan Data/9. FGAE responses.docx]

**Family Guidance Association of Ethiopia (FGAE): cervical cancer activities**

1. Cervical cancer (Cx Ca) precancerous lesion screening was done using VIA and Pap smear. VIA was done by nurses/midwives while a Pap smear was done by a pathologist. Slide smears were done by nurses/midwives. Pap smear costs ETB300 per test. Pap smear tests were also done by Marie Stopes International clinics. But it was found to be more expensive than FGA. About 120-150 Pap smear tests per week were done by FGA. Of which 10 – 14% of the tests turned out to be positive. The Pap smear has been started by FGA in 1990 EC. FGA was known to provide quality Cx Ca precancerous lesion screening services.
2. Cx Ca precancerous lesion treatment was done using Cryotherapy and LEEP. LEEP was done by a gynecologists and cryotherapy by midwives/nurses.
3. HPV DNA test was done by EPHI – a molecular technique.
4. FGA was working with 4 clinics (1 Merkato, 2 Saris, and 1 Meshualekia) in Addis Ababa. However, the location of FGA clinics was found to be a challenge, not easily accessible.
5. Douching is not recommended during the Pap smear test. Due to a lack of awareness of this procedure, patients were forced to go back home and come back afterward for the test. This has contributed to the extended TAT of the test.
6. Stage 3 Cx Ca was either referred back to the health center for further referral to their respective hospitals or referred to St. Paul Hospital or Black Lion Hospital or Zewditu Memorial Hospital by FGA.
7. Scale-up of the FGA services has been done throughout the country.

- The FGA structure is down to the grassroots level throughout the country.
- There were 46 FGA clinics throughout the country, i.e., 10 confidential clinics, 15 medium clinics, 13 your centers, 7 model clinics, and 1 MCH center. These community clinics were evenly distributed throughout the country. These clinics were run by public health officers (BSc), nurses (BSc), and midwives. There was some donor support for the activities done by FGA.
- FGA supports **484 active public health facilities and 354 private clinics** in capacity building, i.e., training and mentoring the professionals.
- There are 8 FGA training centers in the country such as Bahir Dar, Hawassa, Mekelle, Adama, Jimma, HQ, …
- FGA does print and distribution the guidelines.
- FGA does renovation of the health facilities to improve equity of its services in the country.
