# Supplementary material for: Barriers and facilitators to implementation of the Ethiopian national cancer control plan strategies: Implications for cervical cancer services in Ethiopia
Source: PLOS Glob Public Health. 2024 Jul 22;4(7):e0003500. doi: 10.1371/journal.pgph.0003500 (PMC11262691; doi:10.1371/journal.pgph.0003500)
Supplement: S4 File — (ZIP) [file pgph.0003500.s004.zip › Secondary Data/1. Cervical Cancer Performance (July2021 - June2022).docx]

Percentage achievement of HPV vaccination, cervical cancer screening, and treatment in Ethiopia (July 2021 to June 2022)

| # | Activity | # 12-month plan (y) | # Total performance (x) | % achievement (x/y*100) |
| --- | --- | --- | --- | --- |
| 1 | HPV 1 vaccine | 1,114,243 | 1,170,860 | 105% |
| 2 | HPV 2 vaccine | 1,213,224 | 1,015,398 | 83.4% |
| 3 | Screening | 500,000 | 274,756 | 55% |
| 4 | Treatment | 27,000 | 14,213 | 52.64% |

Percentage achievement of HPV vaccination, cervical cancer screening, and treatment in Addis Ababa (July 2021 to June 2022)

| # | Activity | # 12-month plan (y) | # Total performance (x) | % achievement (x/y*100) |
| --- | --- | --- | --- | --- |
| 1 | HPV 1 vaccine | 28,291 | 21,872 | 77% |
| 2 | HPV 2 vaccine | 27,051 | 16,746 | 62% |
| 3 | Screening | 56,620 | 53,726 | 95% |
| 4 | Treatment | 2,433 | 1,683 | 69% |
