# Supplementary material for: Barriers and facilitators to implementation of the Ethiopian national cancer control plan strategies: Implications for cervical cancer services in Ethiopia
Source: PLOS Glob Public Health. 2024 Jul 22;4(7):e0003500. doi: 10.1371/journal.pgph.0003500 (PMC11262691; doi:10.1371/journal.pgph.0003500)
Supplement: S4 File — (ZIP) [file pgph.0003500.s004.zip › Secondary Data/2. NCCP vs other strategies review.docx]

Ethiopia came up with the first National Cancer Control Plan (NCCP) in 2015 which recommends a comprehensive cancer control strategy and interventions with estimates of the cost required to deliver the plan over five years. The plan outlines interventions to reduce the burden of cancer through changes in lifestyle, primary prevention, screening, early diagnosis, appropriate follow-up, treatment, and provision of palliative care (FMoH, 2015). It recognizes the need for multi-sectoral involvement in the fight against cancer. However, the NCCP needed revision considering the latest WHO guidelines and information for the improvised management of cervical cancer in the country. The NCCP linkage with other national existing strategies was reviewed in this study. The NCCP was set in other national strategies to prevent and control cancer in concerted efforts. The key relevant strategic and policy documents reviewed for the link with NCCP were 1) National Health Sector Transformation Plan II (2020-2025), 2) National Strategic Action Plan (NSAP) for control of NCDs, 3) Guideline for Cervical Cancer Prevention and Control, 4) National Reproductive Health Strategy, and 5) A Roadmap for Optimizing the Ethiopian Health Extension Program. Ministry of Health put forward crosscutting cervical cancer interventions within the aforementioned key strategies, plans, and guidelines. The national and regional coordination and leadership of cervical cancer service delivery were managed by the Ministry of Health and the Addis Ababa City Administration Health Bureau, respectively. The major NCCP areas of linkage are indicated in the Table below.

**Table. Summary of the National Cancer Control Plan linkage with other guidelines, plans, and strategies.**

| **#** | **Guidelines/plans/strategies** | **Linkage with the national cancer control plan** |
| --- | --- | --- |
| **1** | Health Sector Transformation Plan of Ethiopia  (2020/21-2024/25) | The overall objective of the Ethiopian health sector transformation plan II (HSTP-II) for 2020-2025 is to improve the health status of the population–by accelerating progress towards universal health coverage, protecting populations during health emergencies, transforming woredas/districts, and improving the health system’s responsiveness. The HSTP II included the NCCP as one of the major healthcare intervention areas to be addressed with the public-private partnership in the country. The HSTP II targets to increase the proportion of women 30-49 years screened for cervical cancer from 5% to 40% (FMoH, 2021). |
| **2** | National Strategic Action Plan (NSAP) for Control of non-communicable diseases in Ethiopia (2014-2016) | In Ethiopia, over 80% of deaths from NCDs are caused by cardiovascular disorders, cancer, diabetes mellitus, and chronic obstructive pulmonary disease (FMoH, 2021). The NSAP considers cancer as one of the four NCDs prevalent in the country. The NCCP aims to augment the implementation of the National Strategic Action Plan for the control of NCDs. NCDs share similar risk factors and prevention mechanisms. The NCCP put forward strategic intervention to reduce exposure to common risk factors including tobacco use, harmful use of alcohol, and exposure to environmental carcinogens for cancer which applies to other NCDs with varying degrees. |
| **3** | Guideline for Cervical Cancer Prevention and  Control (2021) | The NCCP is coordinated by the MOH and other stakeholders for the prevention and control of cancer in Ethiopia. It aims to minimize disability, improve survival rates, and promote a better quality of life for cancer patients. It also aims to maximize the value of resources to reduce physical, financial, and emotional strain on individuals and families suffering from cancer. This is planned to be achieved through public awareness programs, risk identification, screening, early detection, diagnosis, and treatment of cancer. The NCCP provides various strategies, objectives, interventions, and key monitoring indicators for the prevention and control of cancer in Ethiopia. The strategies also incorporated other countries' experiences in the fight against cancer. Similarly, the Guidelines for Cervical Cancer Prevention and Control (2021) guide public awareness, risk identification, HPV vaccination, screening, early detection of pre-cancerous lesions, diagnosis, and treatment of cervical cancer at primary, secondary, and tertiary healthcare levels in Ethiopia. These guidelines are in line with and recognized by the NCCP. |
| **4** | National Reproductive Health Strategy (2016-2020) | The Ethiopian health policy aims to improve the health of women, adolescents, and youth through the availability and provision of quality reproductive health services in a multisectoral approach. The policy requires integrated service delivery of the key interventions including the prevention and treatment of sexually transmitted infections (STIs) and reproductive organ cancers (ROCs). The policy strategies are implemented in partnership with various directorates of the MOH, regional health bureaus, and national and international development partners.  Reproductive organ cancers are among the major cancers affecting women. Human papillomavirus (HPV) is a sexually transmitted infection and the major causative agent for cervical cancer. The sexually transmitted infection (STI) strategies and cervical cancer guidelines aim to provide coordinated services to address HPV through the implementation of nationwide vaccination programs by primary healthcare facilities. |
| **5** | A Roadmap for Optimizing the Ethiopian Health Extension Program (2020-2035) | Primary Health Care Units (PHCUs) encompass the Health Extension Program (HEP). The HEP is a community health program designed to provide 16 “packages” by a team of two Health Extension Workers (HEWs) through prevention and control of diseases, basic hygiene, environmental sanitation, and family health services. The services are delivered in community health posts with a catchment area of 5,000 population. The program was expanded to urban settings in 2009 with the inclusion of non-communicable diseases (NCDs) and community engagement with the women's development team in 2011 for the scale-up of services in the one-to-five network (6 families).  Furthermore, the new HEP strategies were required to address the local health demands considering changes in the country’s epidemiology, demography, and socioeconomic factors. Accordingly, a Roadmap for Optimizing the Ethiopian Health Extension Program (2020-2035) was developed based on the challenges identified through the 2019 national assessment of the HEP to facilitate achieving universal health coverage (UHC) through primary healthcare. The HEP roadmap requires HEWs to disseminate public awareness messages, identify the most at-risk women for cervical precancerous lesions, and forward them to their respective public health centers. |
