# Supplementary material for: Barriers and facilitators to implementation of the Ethiopian national cancer control plan strategies: Implications for cervical cancer services in Ethiopia
Source: PLOS Glob Public Health. 2024 Jul 22;4(7):e0003500. doi: 10.1371/journal.pgph.0003500 (PMC11262691; doi:10.1371/journal.pgph.0003500)
Supplement: Attachment — Submitted filename: PlLoSGH_review.docx [file pgph.0003500.s005.docx]

Overview:

This study combines qualitative and quantitative approaches to examine the facilitators and barriers associated with Implementation of the Ethiopian National Cancer Control Plan in eight facilities in Addis Ababa. Using interviews, secondary data review and surveys, for data collection the study attempts to describe the status of implementation and provides recommendations for improvement. The authors apply thematic analysis for the qualitative data to identify and highlight key gaps, and descriptive analysis for the quantitative data to show how facilities performed in the implementation of cancer control.

Major issues

- There is a mismatch between what the authors introduce the paper as, the methods described, and the results presented. While the main objective was to describe barriers and facilitators, the authors also include facility performance data but fail to demonstrate how these data are meeting the stated objective, and whether they converge or diverge with the analysis of determinants. The write-up could use more details to properly contextualize this work, showing how the methods will address the stated objective, and properly describing the five key policies that frame the research and the author’s inclusion of the performance evaluation.
- The study is described as a mixed methods design, however, the authors do not actually demonstrate the mixing of methods which would require in-depth synthesis of both qualitative and quantitative data to show areas of convergence or divergence. As is, this would be a mixed approaches study not mixed methods design. If mixed methods, please provide rationale for this choice and a citation.
- The cancer control framework is introduced but not described well enough to contextualize the measures that emerge from it. The paper would use a figure summarizing this framework.
- Methods notes that facilities were randomly selected but no details of the randomization process are described.
- In lines 103 -104 the authors introduce a randomly selected group of comparison clinics but fail to provide justification for this choice. Furthermore, neither the results nor the discussion sections reference these “comparison clinics”.
- The description of the qualitative analysis approach is insufficient. How were the qualitative data coded? Please provide more detail.
- What did the survey measure? Describe the variables in the methods to contextualize the results especially from line 153
- Does the author have a statistical analysis plan? How was the survey data analyzed? Please provide an extensive description of how cervical cancer performance was estimated. As is, the methods are lacking in important details of the measures and their definitions. (lines 112-115)
- What aspects of ethical research conduct were applied to the execution of the in-depth interviews?
- Line 119 introduces a rating scale but the authors do not include an explanation/justification for this inclusion. Was there a rating scale for facilitators? If not, why not? If this rating is from published literature, please include a citation.
- Overall, the results could use significant revision to improve flow, organization and to match the proposed objective and methods. Furthermore, the results currently reads like a list. the authors could benefit from summarizing; identify the main points and then use illustrative examples to support. The authors should also be mindful of including a discussion of the results in this section.
- The results are significantly skewed towards barriers. Were there other facilitators? The points about the First lady’s gravitas on Cervical cancer control is discussed in the introduction so would you still include this in the results (lines 131 -135) ? Did you mean to say that from the interviews her role was emphasized?
- The quote included in lines 142-145 does not match the point in the paragraph preceding it.
- Table 2 presents results from 4 facilities. You mentioned that you sampled 8 clinics. Where are data from the rest? Lastly, please edit the column headers to provide information about what the numbers represent.
- Lines 157-159, how dos the MOH report fit into this work? This was not discussed in the methods.
- Line 226 introduces a document review which was not described in the methods.
- Discussion summarizes this work as an examination of challenges but this is not the stated objective at the beginning of the paper.
- The discussion mainly restates the results and could use major revisions to better situate the findings within the broader literature and demonstrate the significance of this work.

Minor issues

- The paper is acronym-heavy, making it difficult to follow. I suggest using fewer acronyms.
- separate data collection and data analysis sections
- was the survey self-administered or interview-assisted?
- Separate the qualitative from the quantitative parts within the data collection and analysis descriptions
- Results: Include at least one quote in the results detailing facilitators
- Separate main ideas using paragraphs
