# Supplementary material for: Barriers and facilitators to implementation of the Ethiopian national cancer control plan strategies: Implications for cervical cancer services in Ethiopia
Source: PLOS Glob Public Health. 2024 Jul 22;4(7):e0003500. doi: 10.1371/journal.pgph.0003500 (PMC11262691; doi:10.1371/journal.pgph.0003500)
Supplement: Attachment — Submitted filename: Response to Reviewers.docx [file pgph.0003500.s006.docx]

**Manuscript Number: PGPH-D-23-00909**

**Response to Reviewers**

**Major issues**

| **Comment** | **Response** | **Revisions made in the manuscript** | **Page** | **Line #** |
| --- | --- | --- | --- | --- |
| There is a mismatch between what the authors introduce the paper as, the methods described, and the results presented. While the main objective was to describe barriers and facilitators, the authors also include facility performance data but fail to demonstrate how these data are meeting the stated objective, and whether they converge or diverge with the analysis of determinants. The write-up could use more details to properly contextualize this work, showing how the methods will address the stated objective, and properly describing the five key policies that frame the research and the author’s inclusion of the performance evaluation. | - We accept your comment. We have now aligned the objective, methods, and results to match. We have provided details showing how the methods will address the study objective and a proper description of the study variables. The title is revised to reflect this, i.e., “Predictors of Ethiopian National Cancer Control Plan Implementation Strategies: Implications for Cervical Cancer Services in Addis Ababa Hospitals.” The predictors (facilitators and barriers) of the national strategies of the cancer control plan encompass the entire health system including the service delivery at health facilities, and users and communities. Hence, this study determined the predictors of implementation strategies of the cancer control plan in light of cervical cancer services or performance at hospitals in Addis Ababa based on scientific principles underpinning the problem phenomenon. The data exploring the strategies and the hospital’s survey were collected simultaneously and analyzed separately but converged for the interpretation of the determinants. |  | 1  3  4-16 | 1-3  74-77  80-311 |
| The study is described as a mixed methods design, however, the authors do not actually demonstrate the mixing of methods which would require an in-depth synthesis of both qualitative and quantitative data to show areas of convergence or divergence. As is, this would be a mixed approaches study not a mixed methods design. If mixed methods, please provide a rationale for this choice and a citation. | - We agree with your recommendation and revised the study design to a “mixed methods approach.” | - The study used a convergent parallel mixed methods approach, whereby qualitative data (key informant interviews), and quantitative data (a survey and a review of secondary sources) were collected concurrently, analyzed independently, and then connected and interpreted. | 4 | 82-84 |
| The cancer control framework is introduced but not described well enough to contextualize the measures that emerge from it. The paper will use a figure summarizing this framework. | - We accept your comment. The cancer control plan’s framework is now better described to indicate the key areas of implementation, strategies, objectives, interventions, and monitoring mechanisms. We have also provided a figure to reflect this. | - The Ethiopian NCCP implementation framework has defined specific strategies, objectives, activities, and monitoring indicators to prevent and control cancer. Fig. 1 outlines our conceptual framework. A continuum of cancer care requires a coordinated array of actions, beginning with public awareness campaigns, risk identification, and HPV vaccination as fundamental preventive measures. This is followed by early cancer detection and screening, diagnostic and treatment services, and palliative care. In addition, the NCCP mandates that the nation establish an ongoing research and surveillance system [6]. | 3 | 62-68 |
| Methods note that facilities were randomly selected but no details of the randomization process are described. | - We agree with your comment and now provided an elaborated process for the selection of hospitals in this study. | - The most recent census of the hospitals was obtained from Addis Ababa City Administration Health Bureau. The sampling frame consisted of five public general hospitals, and two public specialty hospitals which provide cervical cancer services. Then, three public general hospitals (out of five), and one public specialized hospital (out of two) providing oncology services were randomly selected using Microsoft Excel. In addition, three private general hospitals (out of eighteen), and one private specialized hospital (out of two) were randomly chosen to determine the private sector’s cervical cancer services. | 5-6 | 109-116 |
| In lines 103 -104, the authors introduce a randomly selected group of comparison clinics but fail to provide justification for this choice. Furthermore, neither the results nor the discussion sections reference these “comparison clinics”. | - We agree with your comment and revised the selection process for private hospitals. The survey was meant to identify and show the existing cervical cancer service challenges in the both public and private sectors. Hence, the results and discussion sections showed the survey outcomes from public and private health institutions but no comparison was made. We made changes to “comparison clinics” in these sections considering the aforementioned intention of this study. | - In addition, three private general hospitals (out of eighteen), and one private specialized hospital (out of two) were randomly chosen to determine the private sector’s cervical cancer services. Specialty hospitals possessed a minimum bed capacity of 110, were connected to a medical school to provide specialist curative and rehabilitative services, and had modern diagnostic and treatment facilities. In contrast, general hospitals offered obstetrics and gynecological services along with preventive, curative, rehabilitative, and promotive treatments and had a minimum of 50 beds. The aforementioned requirements were also considered in the selection process of both public and private hospitals in this study. | 5-6 | 114-121 |
| The description of the qualitative analysis approach is insufficient. How were the qualitative data coded? Please provide more details. | - We agree with your observation and now provided more details on the qualitative data analysis approach. | - Amharic transcriptions and English translations were made of the key informant interviews' audio recordings. The English translations of the key informant interview textual data and notes taken during interviews were arranged and categorized in Microsoft Excel, coded, and analyzed into six pre-established themes (political commitment, priority setting, interagency cooperation, evidence-based practice, population empowerment, and incentives), and sixteen sub-thematic areas using a thematic analysis approach. To represent different ideas or concepts, data segments were systematically given labels as part of the coding process for qualitative data. The codes were subsequently put together for each of the themes. | 7-8 | 158-166 |
| What did the survey measure? Describe the variables in the methods to contextualize the results, especially from line 153 | - We have now included the variables measured in the survey in the methods section (under “data collection procedure”) and linked them with the results section. | - A checklist was used for the extraction of data on screening for cervical cancer achievement (tests versus annual plan) by examining the hospitals’ cervical cancer registers (for July 1, 2021, to June 30, 2022). - Examine the accessibility and availability of leadership, information, financing, medicines, diagnostics, and testing, as well as human resources and service delivery. | 7  7 | 154-156  146-147 |
| Does the author have a statistical analysis plan? How was the survey data analyzed? Please provide an extensive description of how cervical cancer performance was estimated. As is, the methods are lacking in important details of the measures and their definitions. (lines 112-115) | - We agree with your observation and now we have included the procedures on how the statistical analysis of cervical cancer performance was determined under the “Data processing and analysis” section. Important details of the measures and their definitions are included as well. |  | 7-8 | 157-182 |
| What aspects of ethical research conduct were applied to the execution of the in-depth interviews? | - We have now provided more details on the “ethical considerations” applied during the key informant interviews. | - The "consent form and respondent information" were explained to the participants, and any inquiries were properly addressed. Before the interview procedure started, participants were required to read and sign the consent form. Further, the participants received a comprehensive explanation of every question asked during interviews, a component of the study's guidelines. Participants were free to change their minds and discontinue participation at any point during the study. | 9 | 187-192 |
| Line 119 introduces a rating scale but the authors do not include an explanation/justification for this inclusion. Was there a rating scale for facilitators? If not, why not? If this rating is from published literature, please include a citation. | - We agree with your comment. We have now provided the rationale for the inclusion of a rating scale for the identified challenges. The focus was on “areas of improvement” based on the “challenges” not on “facilitators”. To facilitate this, we have consulted the reference: WHO. Better non-communicable disease outcomes: challenges and opportunities for health systems: assessment guide. Copenhagen: WHO Regional Office for Europe; 2014 [11]. | - The classification of the identified challenges was carried out using a scale of 1=Minor, 2=Moderate, 3=Major, and 4=Major persistent [11]. This could help the decision-makers to focus, prioritize, and invest on identified issues of cervical cancer services. | 8 | 176-179 |
| Overall, the results could use significant revision to improve flow, organization and to match the proposed objective and methods. Furthermore, the results currently read like a list. The authors could benefit from summarizing; identifying the main points and then using illustrative examples to support them. The authors should also be mindful of including a discussion of the results in this section. | - We have made major revision of the results section to improve its structure, flow, and organization based on the objectives and methods of the study. The subsections included 1) Key informant interviews on the NCCP strategies, 2) Cervical cancer services attainment at the national level, 3) The NCCP linkage with other strategies, and 4) Cervical cancer service delivery at hospitals. We have also provided a discussion of the results and summarized the identified key points. |  | 9-16 | 195-311 |
| The results are significantly skewed towards barriers. Were there other facilitators? The points about the First Lady’s gravitas on Cervical cancer control are discussed in the introduction so would you still include this in the results (lines 131 -135)? Did you mean to say that from the interviews her role was emphasized? | - Apart from the First Lady’s strong support of cancer control the key informant interviews also identified other facilitators for the NCCP implementation including the presence of the national technical working group, community-based health insurance, deployment of middle-level oncology professionals, a decentralized health system, and availability of the cancer guidelines at health facilities. The Ethiopian First Lady’s guidance on cervical cancer control is included in the results section rather than in the introduction. Her role was highlighted during the interviews with participants. | - Ethiopia's First Lady and Minister of Health, who co-chair the National Cancer Committee, which coordinates cancer prevention and control activities, was a significant facilitator in the NCCP's implementation. The presence of the National Technical Working Group (TWG), the use of community-based health insurance, the deployment of middle-level oncology professionals, a decentralized health system, the availability of cancer guidelines, and the implementation of the combat cervical cancer (3Cs) initiative were all identified as facilitators as well. | 11 | 205-210 |
| The quote included in lines 142-145 does not match the point in the paragraph preceding it. | - We will ensure that the quote is placed under the right paragraph. However, we intended to show that there was inadequate coordination including funding among the Addis Ababa City Administration Health Bureau and other government agencies such as the Addis Ababa Education Bureau, Women, Youth, and Child Affairs Bureau, and Sports Bureau as well as through the private sector. | - “There was some liaison with Addis Ababa City Administration Education Bureau (AACAEB). However, this area was not adequately exploited and it needs to be strengthened with AACAEB, Women, Youth, and Child Affairs, and Sports Bureau, and others.” (KII 9 in AACAHB) | 12 | 232-235 |
| Table 2 presents results from 4 facilities. You mentioned that you sampled 8 clinics. Where are the data from the rest? Lastly, please edit the column headers to provide information about what the numbers represent. | - Yes, Table 2 shows results from 4 facilities because the VIA test, HPV DNA test, and Colposcopy were done in the 4 government hospitals only. While the 4 private facilities were providing Pap smear tests only. The information on the column headers in Table 2 is now edited and changed to Table 5. | - Tablet 5. Percentage achievement of cervical cancer screening at public hospitals in Addis Ababa (n=4). | 16 | 298-299 |
| Lines 157-159, how does the MOH report fit into this work? This was not discussed in the methods. | - We agree with your observation. We have now discussed this in the methods section to fit the results section. | - The principal investigator conducted secondary data reviews at the Ministry of Health to assess target attainment (performance per the annual plan) for HPV vaccination, screening, and treatment (from July 1, 2021, to June 30, 2022). The Ministry of Health's cervical cancer register report provided data on the number of women who were screened for the disease and who underwent treatment in the country. While the national EPI register reports were used to determine the HPV vaccination target attainment. | 6-7 | 134-139 |
| Line 226 introduces a document review which was not described in the methods. | - We concur with your comment. We have now described the document review points in the methods section. | - The interventions within the cancer control plan and other strategies that are included in the cervical cancer guidelines, health extension roadmap, health sector transformation plan, strategic plan for non-communicable diseases, and reproductive health strategy were examined through document reviews. | 7 | 139-142 |
| Discussion summarizes this work as an examination of challenges but this is not the stated objective at the beginning of the paper. | - We have now included the key barriers and facilitators in the objective of the study, and the discussion section of the manuscript |  | 3  17-23 | 74-77  312-451 |
| The discussion mainly restates the results and could use major revisions to better situate the findings within the broader literature and demonstrate the significance of this work. | - We have now made major revisions in the discussion section to better reflect the main results of the study by minimizing repetitions. Efforts are made to relate them to the available literature and demonstrate the significance of this work. |  | 17-23 | 312-451 |

**Minor issues**

| **Comment** | **Response** | **Revisions made in the manuscript** | **Page** | **Line #** |
| --- | --- | --- | --- | --- |
| The paper is acronym-heavy, making it difficult to follow. I suggest using fewer acronyms. | - We agree with your suggestion. We have now minimized the number of acronyms to make it easy to follow. |  | 1-24 | 1-479 |
| Separate data collection and data analysis sections. | - We agree with your recommendation. We have now separated the data collection and analysis sections. |  | 6-7  7-8 | 122-156  157-182 |
| Was the survey self-administered or interview-assisted? | - The survey was face-to-face interview-assisted. |  | 7 | 153 |
| Separate the qualitative from the quantitative parts within the data collection and analysis descriptions | - We accept your comment. We have now separated the qualitative and quantitative parts in the data collection and analysis sections. |  | 6-7  7-8 | 122-156  157-182 |
| Results: Include at least one quote in the results detailing the facilitators | - We accept your recommendation. A quote on facilitators is now included in the results section. |  | 11 | 211-215 |
| Separate main ideas using paragraphs | - Your suggestion is considered and the main ideas are now separated using paragraphs. |  | 1-24 | 1-479 |
