# Supplementary material for: Barriers and facilitators to implementation of the Ethiopian national cancer control plan strategies: Implications for cervical cancer services in Ethiopia
Source: PLOS Glob Public Health. 2024 Jul 22;4(7):e0003500. doi: 10.1371/journal.pgph.0003500 (PMC11262691; doi:10.1371/journal.pgph.0003500)
Supplement: Attachment — Submitted filename: Response to Reviewers.docx [file pgph.0003500.s007.docx]

**Manuscript Number: PGPH-D-23-00909R1**

**Response to Reviewers**

**Academic editor comments**

| **Comment** | **Response** | **Revisions made in the manuscript** | **Page** | **Line #** |
| --- | --- | --- | --- | --- |
| Title | - We accept your suggestion and the title is now changed to reflect the contents of the article. The term "predictors" is eliminated from the title, and throughout the manuscript due to its connotation of causal inference. | The title is changed to “Barriers and facilitators to implementation of the Ethiopian National Cancer Control Plan strategies: Implications for cervical cancer services in Ethiopia”. | 1 | 1-3 |
| Abstract | - We accept your observation and the strategies considered in the study are now included in the Abstract section. Moreover, we focused on barriers to the implementation of strategies throughout the document. | Strategies that are included in the manuscript are “political commitment, priority setting, interagency cooperation, evidence-based practice, population empowerment, and incentive systems”. | 1 | 16-17 |
| Methods and results | - We agree with your recommendation that it is helpful to eliminate the quantitative survey component as it adds more confusion than value to the research article. Also, the quantitative results require more description to show a better linkage with the qualitative part. Consequently, the quantitative survey part is eliminated from the “Methods and Results” section to avoid confusion in presenting the quantitative aim and connecting it with the qualitative work. - We comply with your comment on the outcome of quantitative or statistical analyses. The examination of secondary sources is a qualitative synthesis rather than a quantitative analysis. The study aligns more with qualitative research or constitutes a narrative synthesis of data from secondary sources and registers, providing a descriptive presentation of proportions or a summary of national health reports/records. | Instead of “a convergent parallel mixed-methods approach”, “a qualitative method approach” is maintained under “Study design and period”.  Only “qualitative analysis” is provided under “Data processing and analysis.” | 4  6 | 83-84  128-141 |
| Other comments | - We agree with your comments. We have removed specific political personalities such as “the First Lady” to avoid the appearance of a political agenda/manifesto from the manuscript and included your suggested words. - We have tried to provide objective and citable statements throughout the study. | We have included "political figures," "key female decision makers," or "influential people”. | 1  7  9  13  18  19 | 22-23  157  162  257  374  380 |
| References | - We agree with your comment. The references used in the “mixed methods approach” are now eliminated. | Only the references for a “qualitative study” are included in the manuscript. | 20-22 | 410-501 |

**Reviewers' comments**

| **Comment** | **Response** | **Revisions made in the manuscript** | **Page** | **Line #** |
| --- | --- | --- | --- | --- |
| Reviewer #2: The authors have made improvements to the manuscript. However, the storyline is still not clear enough and the writing still requires close editing. As is, the paper is still disjointed. There are multiple data sources and results that are not providing a cohesive main message and certain parts of the results don't seem to inform each other. It is also unclear what the denominators in the results are. I would recommend excluding the quantitative data. The comparison of policies could use more nuanced reporting and better contextualization in the introduction and methods. | - We agree with your point. We have now excluded the quantitative data. The manuscript describes methodologically and ethically rigorous research with conclusions that are appropriately drawn based on qualitative data. Consequently, we have made the necessary edits in various parts of the manuscript. We have tried to integrate multiple data sources including key informant interviews, data from secondary sources and registers, and document reviews to inform each other and provide a unified and consistent key message. - The denominators mainly for cervical cancer services are made clear. - To better understand the linkage of NCCP with other national policies, we have provided a better contextualization in the “Introduction”, and “Materials and Methods” sections. |  | 1-22  11-12  3  4  5  6 | 1-501  228-235  76-78  87-89  103-105  123-127 &  139-141 |
| Reviewer #3: The Authors did a wonderful job addressing the reviewers’ comments in detail. The one aspect that I still feel needs revision is the description/use of mixed methods. If the authors would like to stick with the description of convergent parallel design, then the results need to be integrated in some way. Do the qualitative and quantitative results relate to one another? Do they confirm or disprove one another? Did any of the qualitative results provide context to the quantitative findings? OR if the authors would like to keep the results and discussion as is, then I recommend saying that multiple methods (quantitative and qualitative) were used, instead of mixed methods. | - We agree with your comment. We have now removed the description of “the convergent parallel mixed-method” approach. Only a “qualitative method approach” is maintained. - We have revised the manuscript by eliminating the quantitative part and retaining the qualitative study only. |  | 4  1-22 | 83-84  1-501 |
