# Supplementary material for: Barriers and facilitators to implementation of the Ethiopian national cancer control plan strategies: Implications for cervical cancer services in Ethiopia
Source: PLOS Glob Public Health. 2024 Jul 22;4(7):e0003500. doi: 10.1371/journal.pgph.0003500 (PMC11262691; doi:10.1371/journal.pgph.0003500)
Supplement: Attachment — Submitted filename: Response to Reviewers.docx [file pgph.0003500.s008.docx]

**Manuscript Number: PGPH-D-23-00909R2**

**Response to Reviewers**

| **Comment** | **Response** | **Revisions made in the manuscript** | **Page** | **Line #** |
| --- | --- | --- | --- | --- |
| Please review your reference list to ensure that it is complete and correct. If you have cited papers that have been retracted, please include the rationale for doing so in the manuscript text, or remove these references and replace them with relevant current references. Any changes to the reference list should be mentioned in the rebuttal letter that accompanies your revised manuscript. If you need to cite a retracted article, indicate the article’s retracted status in the References list and also include a citation and full reference for the retraction notice. | - We concur with your recommendation. We reviewed our reference list to ensure that it is complete and correct. In addition, we checked for any cited papers that might have been retracted. - We have included a new reference for COREQ (Ref. #11). - Reference #29. We have replaced “WHO. WHO \| Guidelines for screening and treatment of precancerous lesions for cervical cancer prevention; 2013. <https://www.who.int/reproductivehealth/publications/cancers/screening_and_treatment_of_precancerous_lesions/en/>” with the current reference. | - Reference [11] - Reference # 11. Tong A, Sainsbury P, Craig J. Consolidated criteria for reporting qualitative research (COREQ): a 32-item checklist for interviews and focus groups. International Journal for Quality in Health Care. 2007;19(6):349–57. - Reference #29. WHO guideline for screening and treatment of cervical pre-cancer lesions for cervical cancer prevention, second edition. Geneva: World Health Organization; 2021. Licence: CC BY-NC-SA 3.0 IGO. <https://www.who.int/publications-detail-redirect/9789240030824>. | 4  20  22 | 90  434-436  490-492 |
| In the abstract and throughout, instead of referring to “influential females/ women,” please broadly refer to “lack of political support.” | - We respect your comment. Please note that the Ethiopian government was committed to the fight against cancer. However, our findings showed that the support from sub-national levels and charismatic people and celebrities was inadequate though not lacking. | In the Abstract and throughout the manuscript, instead of “influential females/ women” we have referred to:   - inadequate political support - a demand for more political support and its translation to sub-national levels - inadequate comprehensive political support - inadequate political support - insufficient multilayered political support | 1  7  7  13  19 | 28  163-164  166  254  380 |
| Please include the COREQ guideline checklist for qualitative studies in your files and ensure all the requirements of the checklist are met. | - We agree with your recommendation. We have included the COREQ guideline checklist for qualitative studies in our files and ensured all the checklist requirements are met. |  | 4-7 | 81-146 |
| In the Methods, please indicate that the reporting of the study was done in compliance with “the Consolidated criteria for reporting qualitative research (COREQ)” guidelines for reporting qualitative studies. | - We agree with your suggestion. Per your recommendation, under the Methods section, we have included “the consolidated criteria for reporting qualitative research (COREQ)” guidelines for reporting qualitative studies. | The study was reported in compliance with “the consolidated criteria for reporting qualitative research (COREQ)”: a 32-item checklist to assess qualitative studies. | 4 | 89-90 |
| Line 257, please remove “other” in “other high-profile women” | - We accept your comment and removed “other” in “other high-profile women”. | We removed “other” in “other high-profile women” | 13 | 261 |
| Under limitations, please include limitations that are inherent to the study design itself. | - Per your recommendation, we have included limitations that are inherent to the study design itself. | Moreover, social desirability bias might have prevented us from gathering more information. Also, if expert panel discussions had been incorporated into the study design, a diverse range of views would have been collected from participants. | 18 | 369-372 |
| The conclusion, as it is, reads like a truncation of the results and discussion section. Please synthesize and condense into a concise summary (reduced by half) that ties the themes and takeaways into one or two recommendations for a thought-provoking conclusion. | - We agree with your comment. We have revised and made the conclusion succinct to tie the results and discussions within the themes with takeaway recommendations for a thought-provoking ending. |  | 18-19 | 374-389 |
