# Supplementary material for: Barriers and facilitators to implementation of the Ethiopian national cancer control plan strategies: Implications for cervical cancer services in Ethiopia
Source: PLOS Glob Public Health. 2024 Jul 22;4(7):e0003500. doi: 10.1371/journal.pgph.0003500 (PMC11262691; doi:10.1371/journal.pgph.0003500)
Supplement: Attachment — Submitted filename: Response to Reviewers.docx [file pgph.0003500.s009.docx]

**Manuscript Number: PGPH-D-23-00909R3**

**Editor Comments**

| **Comment** | **Response** | **Revisions made in the manuscript** | **Page** | **Line #** |
| --- | --- | --- | --- | --- |
| Please submit your COREQ checklist as part of the supplementary files, not as an email attachment. In the checklist, please refer to headings/ sub-headings and paragraph numbers - do not refer to line numbers as they may change during the production of the paper. | - We agree with your recommendation. We have now submitted the completed COREQ checklist as part of the supplementary files. In the checklist, we referred to headings/sub-headings and paragraph numbers. | - S2 File. Completed consolidated criteria for reporting qualitative studies (COREQ): a 32-item checklist. | 20 | 402- 403 |
| In the ethics statement in the Methods, you have specified that verbal consent was obtained. Please provide additional details regarding how this consent was documented and witnessed, and state whether this was approved by the IRB. | - Please note that verbal consent refers to the verbal version of the consent form. For this we have included in the manuscript the "consent form and respondent information" was explained to the participants, and any inquiries were properly addressed. Then, participants were asked to read and sign a written informed consent form before the interview. This was done per the “Respondent information and consent document” approved by the IRB. | - The "consent form and respondent information" was explained to the participants, and any inquiries were properly addressed. Then, participants were asked to read and sign a written informed consent form before the interview. | 7 | 160-162 |
| Please review your reference list to ensure that it is complete and correct. If you have cited papers that have been retracted, please include the rationale for doing so in the manuscript text, or remove these references and replace them with relevant current references. Any changes to the reference list should be mentioned in the rebuttal letter that accompanies your revised manuscript. If you need to cite a retracted article, indicate the article’s retracted status in the References list and also include a citation and full reference for the retraction notice. | - We accept your recommendation. We reviewed our reference list to ensure that it is complete and correct. In addition, we checked for any cited papers that might have been retracted. We verified that the reference list is complete and correct. No cited paper in the manuscript has been retracted. |  | 20-23 | 420-513 |
