# Supplementary material for: Barriers and facilitators to implementation of the Ethiopian national cancer control plan strategies: Implications for cervical cancer services in Ethiopia
Source: PLOS Glob Public Health. 2024 Jul 22;4(7):e0003500. doi: 10.1371/journal.pgph.0003500 (PMC11262691; doi:10.1371/journal.pgph.0003500)
Supplement: Attachment — Submitted filename: Response to Reviewers.docx [file pgph.0003500.s010.docx]

**Manuscript Number: PGPH-D-23-00909R5**

**Editor Comments**

| **Comment** | **Response** | **Revisions made in the manuscript** | **Page** | **Line #** |
| --- | --- | --- | --- | --- |
| 1. Please add a full list of legends for all your Supporting Information files after the References list. | - We comply with your request. We have now included a full list of legends for all our Supporting Information files after the References list. | - A full list of legends for all our Supporting Information files. | 23 | 509-516 |
